# Mucus-derived biomaterial dressings: a novel approach to accelerate wound healing

**DOI:** 10.7150/thno.115988

**Published:** 2025-07-24

**Authors:** Xuanqi Peng, Ziyi Wang, Leo Wang, Weiliang Hou

**Affiliations:** 1 The State Key Laboratory of Mechanism and Quality of Chinese Medicine, Institute of Chinese Medical Sciences, University of Macau, Macau 999078, China.; 2 Kitsilano Secondary School, 2706 Trafalgar Street, Vancouver, V6K 2J6, Canada.; 3 Department of Gastroenterology, Shanghai Institute of Pancreatic Diseases, National Key Laboratory of Immunity and Inflammation, Changhai Clinical Research Unit, Changhai Hospital, Naval Medical University, Shanghai, 200433, China.; 4 Shanghai Collaborative Innovation Center of Endoscopy, Endoscopy Center and Endoscopy Research Institute Zhongshan Hospital, Fudan University, Shanghai, 200433, China.

**Keywords:** mucus, wound healing, adhesion, natural biomaterial, regeneration

## Abstract

Wound management remains a clinical challenge due to the complexity of healing processes. Traditional dressings with passive protection mechanisms and modern synthetic alternatives often fail to recapitulate the dynamic biological interactions in the wound microenvironment. Mucus is a naturally widely available biomaterial, exhibiting superior bioactive properties as a viscoelastic gel-like substance. Notably, natural mucus derived from diverse biological sources has garnered significant attention as advanced wound dressings. This review explores the potential of natural mucus from animals, plants, microorganisms, and other complex sources as multifunctional wound healing platforms. By analyzing the therapeutic effects of natural mucus, we evaluate its key molecular mechanisms and performance metrics against clinical wound dressings. This establishes a scientific framework for mucus-inspired biomaterials design. The comprehensive assessment not only reveals the untapped potential of renewable biological resources in developing eco-friendly, high-performance wound care alternatives but also provides theoretical guidance for developing next-generation dressings with bioactive, self-adaptive, and environmentally responsive characteristics.

## 1. Introduction

Skin tissue injuries spanning acute trauma to chronic pathologies constitute a global healthcare crisis, with over 5 million annual deaths attributed to wound-related complications 1. Particularly alarming is their status as a leading mortality factor for individuals under 45 years, surpassing many infectious diseases in socioeconomic impact 2. Traditional wound management strategies often lead to fibrotic scarring, surgical site contracture, prolonged healing time and high infection risk 3. An optimal wound dressing should orchestrate all healing phases and maintain physiological homeostasis 4. Current clinical adhesives have some critical limitations. The strong adhesive cyanoacrylates (CAs) have slow degradation and cytotoxic side effects. Fibrin adhesives show weaker adhesion, restricting their application 5. Thus, novel wound dressings must combine adequate adhesion, high biocompatibility and optimal therapeutic efficacy.

**Figure 1:**
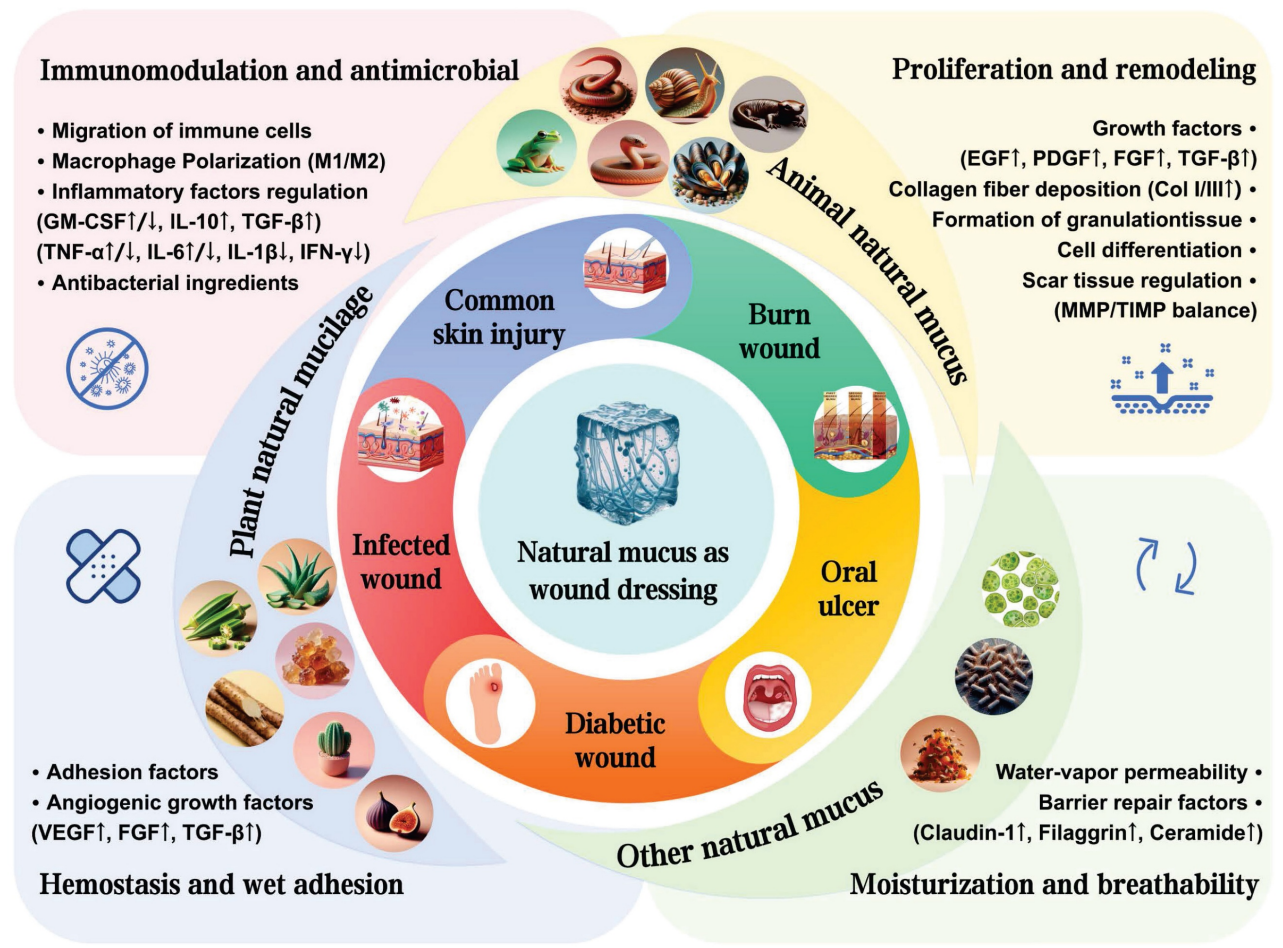
Schematic illustration of natural mucus in the treatment of diverse wound healing models. Created with BioRender.com.

Natural mucus is a viscoelastic bio-secretion typically composed of water, mucin, polysaccharides, lipids, and other bioactive components. Its nonlinear rheological behavior mainly stems from entangled mucin glycoproteins forming transient polymer networks 6. Serving as a multifunctional interface, it can mediate adhesion, lubrication, hydration, and antimicrobial defense in vital movement. Animal mucus is typically secreted by goblet cells in the mucosal layer. In contrast, plant mucilage is generally secreted by seed coats or specialized mucilage glands 7. Microorganisms in nature, such as myxobacteria and microalgae, can also secrete mucus, which holds broad application potential in industrial and biomedical fields 8. Over the past 30 years, research into natural mucus from diverse sources and its therapeutic potential has grown significantly. Various animals and plants secrete sticky tissue fluids, aiding in self-defense, locomotion, and prey capture. For instance, marine mussels anchor themselves to surfaces by secreting viscous proteins, withstanding the enormous shear forces of ocean waves 9. When encountering predators, slugs secrete mucus to adhere on rocky surface to crawl against gravity 10. Geckos possess sticky toe pads to crawl against gravity 11. Snails secrete mucus with lubricating and adhesive properties, maintaining contact with smooth surfaces while crawling 12. Okra mucilage prevents borer insects from entering the interior and consuming the seeds, also providing essential nutrients for growth 13. Cacti mucilage forms a protective film to prevent water evaporation and store nutrients, hence enduring harsh environmental conditions 14. The advantageous properties of natural mucus stem from its molecular composition. Most biomacromolecules in natural mucus are polymers characterized by long linear chains of repeating units, such as chitosan, alginate, hyaluronic acid, dextran, and natural proteins like fibrin and elastin 15. After extraction and freeze-drying, natural mucus can be rehydrated to form natural hydrogels. This complex adhesive system is constructed by covalent bonds and non-covalent interactions 16. Inspired by these natural phenomena, bioadhesives derived from living organisms, natural mucus, exhibit significant potential as the substitutes of traditional wound healing dressings (Figure 1).

**Figure 2:**
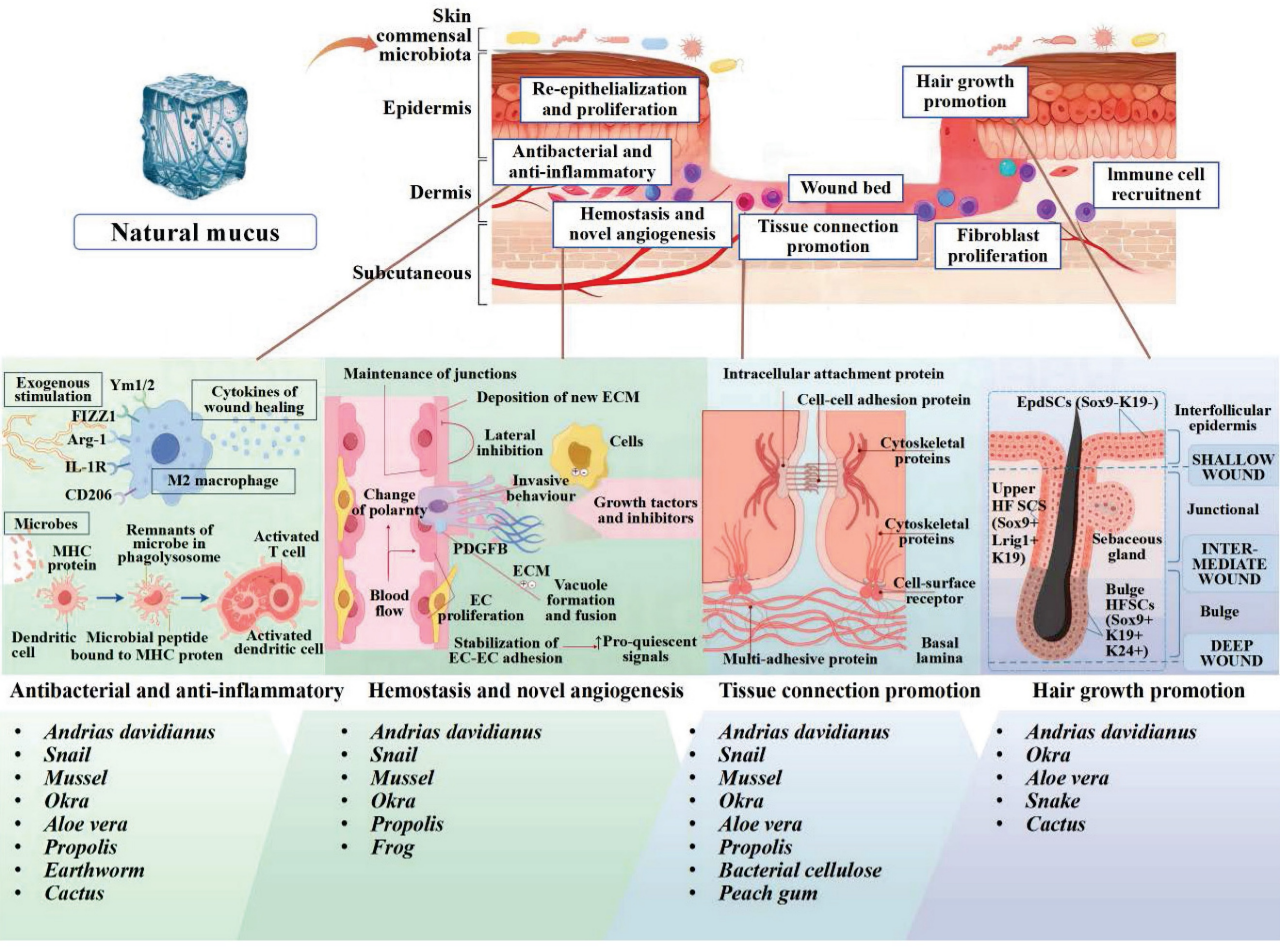
Summarizing the wound healing mechanisms of natural mucus. Created with BioRender.com.

Recently, natural mucus has presented significant advances in the medical, cosmetic and food industries. However, comprehensive summaries and analyses on the specific roles and applications of natural mucus in the wound healing process are still lacking. In this review, we summarized the wound healing properties, cross-linking effects and biochemical functions of natural mucus sourced from various organisms (Figure 2). We also highlighted the application of natural mucus in different types of wound healing models. Furthermore, we analyzed potential issues in wound healing applications and discuss the challenges to clinical adoption, aiming to promote further research into natural biomaterials in wound healing.

## 2. Natural Mucus in Wound Healing Process Regulation

**Figure 3:**
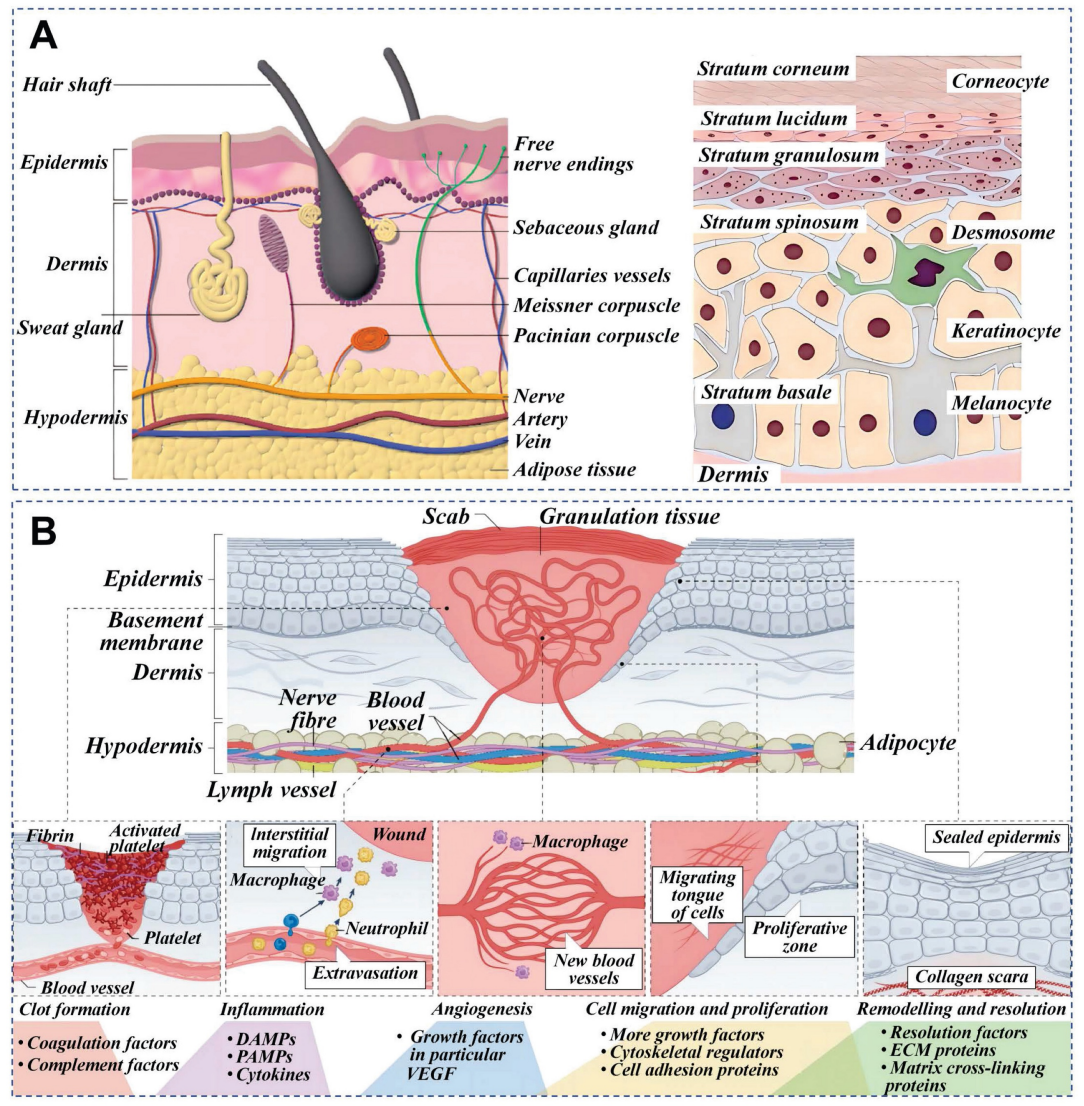
Skin cross-section and wound healing phases. A) Cross-sectional anatomy of skin. Reproduced with permission 21. Copyright 2023, John Wiley and Sons. B) Principal stages of wound healing and evaluation criteria at different wound healing stages. Reproduced with permission 19. Copyright 2024, Springer Nature.

The skin is the largest organ of the human body, composed of epidermis, dermis, and subcutaneous tissue. Figure 3 depicts the epidermis, the outermost layer of the skin, which is often stratified into four distinct layers: the basal layer, spinous layer, granular layer, and horny layer. The basal layer harbors stem cells, which act as the progenitors for keratinocytes, initiating a process of epidermal renewal. As these keratinocytes ascend through the layers, they become adhered by desmosomes within the spinous layer. Keratohyalin granules in the granular layer and dead keratinocytes in the horny layer form a protective barrier against external microorganisms and substances 17. The natural moisturizing factors in the horny layer play a significant role in skin hydration, softness, and elasticity. Natural mucus contains abundant adhesion molecules that strengthen adherent junctions. The dermal-epidermal junction plays an essential role in nutrient transport and immune isolation 18. The dermis is primarily composed of fibroblasts accompanied by nerves, blood vessels, lymphatics, muscles, hair follicles, sebaceous glands, and sweat glands. These fibroblasts are responsible for the synthesis of collagen, elastin, and various enzymes, all of which contribute to the skin's mechanical strength, elasticity, and physiological processes 19. Natural mucus-derived dressings interact with skin microstructure through conserved molecular mechanisms, achieving therapeutic effects via synergistic interactions between key biomolecules and dynamic adaptive mechanisms. Wound healing requires the synchronized activities of inflammation, cell migration, proliferation, matrix deposition, remodeling, and angiogenesis 20. This reparative process can be divided into three distinct yet interwoven and successive stages: inflammation, proliferation and remodeling.

During the inflammatory phase, the evaluation criteria include the appropriateness of the inflammatory reaction, the recruitment and activity of leukocytes, and the balance of pro-inflammatory mediators. The cytokines released by the damaged blood vessel cause the inflammatory response to recruit immune cells. Neutrophils, as the first immune responders, combat infections by phagocytosis and release of antimicrobial factors 22. Macrophages release cytokines and proteases to promote tissue repair 23. Adhesion components like lectins in natural mucus can rapidly exert hemostatic and wound closure effects. In the initial contact with wounds, nature mucus can quickly release a variety of growth factors, enhancing the recruitment of stem cells. Cytokines like TNF, IL-1, and IL-6 are activated to regulate the inflammatory sequelae. Meanwhile, antimicrobial peptides and flavonoids in natural mucus have good antibacterial effects, reducing the risk of wound infection.

During the proliferation phase, the evaluation criteria focus on fibroblast activity, collagen and elastin synthesis and vascularization. These processes are crucial for skin toughness and elasticity. Cytokines such as TGF-β, EGF, PDGF, GM-CSF, and FGF interact with each other to promote matrix synthesis and cell proliferation 24. Mucin in animal mucus can typically interact with dermal tissue to form a dynamic responsive network structure of reversible hydrogen bonds and disulfide bonds. This network can adaptively adjust adhesion via conformational changes in moist environments. Concurrently, proteoglycans and glycosaminoglycans are vital for cell proliferation, movement, differentiation, adhesion, and fiber formation. Plant-derived polysaccharides and pectin components are known to protonate carboxyl groups in acidic wound environments, enhancing electrostatic binding with collagen. These natural mucus substances can simulate the extracellular matrix (ECM) of dermal cells. While their self-adaptive properties can facilitate cell migration and proliferation, thereby accelerating the formation of granulation tissue 25.

During the remodeling phase, focus shifts to collagen reorganization and maturation, vascular network stabilization and the reduction of cell signaling. During this stage, tissue repair is nearing completion and the function is restored. It has been confirmed that natural mucus contains a variety of growth factors associated with wound regeneration, angiogenesis, and epithelial regeneration, such as VEGF, PDGF, EGF, HGF, and bFGF. These growth factors can accelerate wound healing by meeting the remodeling needs of the wound 20. In addition, some special components have also been proven to be effective in this stage. A novel glycosaminoglycans derived from the skin secretion of *Andrias davidianus* (SAGs) can modulate the gene expression related to glycolysis and lipid metabolism in macrophages via the PPARγ pathway, thereby promoting the transition of reparative macrophages. SAGs can also control the proportion of reticulum fibroblasts to curb collagen overexpression, thereby promoting hair follicle regeneration and scarless wound healing 26. The key components of natural mucus are classified according to their biological functions in Table 1.

Dynamic interactions between these components in nature mucus are critical for wound healing (Table 1). Wound healing process could encompass a complex interplay among various cell types, biomolecules and tissues. A comprehensive grasp of the dynamic mechanisms governing cellular and molecular crosstalk provides the foundation for next-generation dressing design 31. Functionally, natural mucus components can be categorized into several categories that work together to coordinate repair processes: structural molecules to providing mechanical support, signaling molecules to mediating cellular responses, hemostatic agents to controlling bleeding, antimicrobial agents to preventing infection, and immunomodulatory molecules to regulating inflammatory cascades. Given the challenges related to clinical wound dressings, nature mucus dressings with innate bioactivity and environmental responsiveness might be a viable and promising alternative.

## 3. Animal-Derived Natural Mucus and Their Properties

Animal mucus, a complex aqueous fluid secreted by goblet or mucous-producing cells lining the epithelial surfaces of organs exposed to the external environment, exhibits viscoelastic, lubricating, and hydration properties due to its composition and structure. These properties are attributed to the glycoprotein mucin, combined with electrolytes, lipids, and other smaller proteins. The structural and functional components of natural mucus offer a robust framework for wound healing 32. And the chemical constituents, functionalities, and properties of animal mucus are influenced by the species of origin, tissue type, secretion method, and environmental conditions 33. Water is the primary component, accounting for approximately 95% of the total mass. This elevated water content endows the mucus with fluidity and lubricative properties 34. Highly branched mucus glycoproteins typically constitute less than 5% of the mucus. The mucin granules, which fuse with the plasma membrane and release upon activation, provide a dynamic and responsive system for wound dressing applications 35. Other components, including lipids, inorganic salts, electrolytes, and antimicrobial substances such as lysozyme and immunoglobulins, account for about 1%. These components regulate the osmotic pressure, pH, antimicrobial and anti-inflammatory properties of the mucus 36.

In this section, the design concepts of mucus from different animal sources are discussed as natural wound-healing dressings (Table 2). We emphasize the adhesion mechanisms and preparation methods of mucus derived from *Andrias davidianus*, snails, and mussels for natural wound-healing dressings, such as innate adhesiveness, anti-inflammatory, and biocompatible properties.

### 3.1. Andrias davidianus mucus

*Andrias davidianus* belongs to the Cryptobranchidae family originating from China. This species can attain lengths of 1 to 2 meters and typically weigh between 20 and 25 kilograms 83. Its huge skin surface is uniformly populated with numerous granular glands and mucous glands. These skin glands secrete a mixture of milky-white and watery transparent liquids when stimulated, forming the skin secretions *Andrias davidianus* (SSAD), a mucus composite with distinctive bioactive properties. Contemporary research indicates that non-invasive sampling methods, such as mechanical or electrical stimulation, can enhance the efficiency of SSAD 84. This natural advantage provides a pathway for green development and sustainable utilization in harnessing the biological resources of *Andrias davidianus*. Utilizing a combination of two-dimensional gel electrophoresis and mass spectrometry techniques, 155 proteins have been identified in the *Andrias davidianus* mucus 85. Subsequent gene ontology analysis indicates that these proteins are implicated in ECM organization, defense responses, immune reactions, wound healing, and respiratory processes.

**Figure 4:**
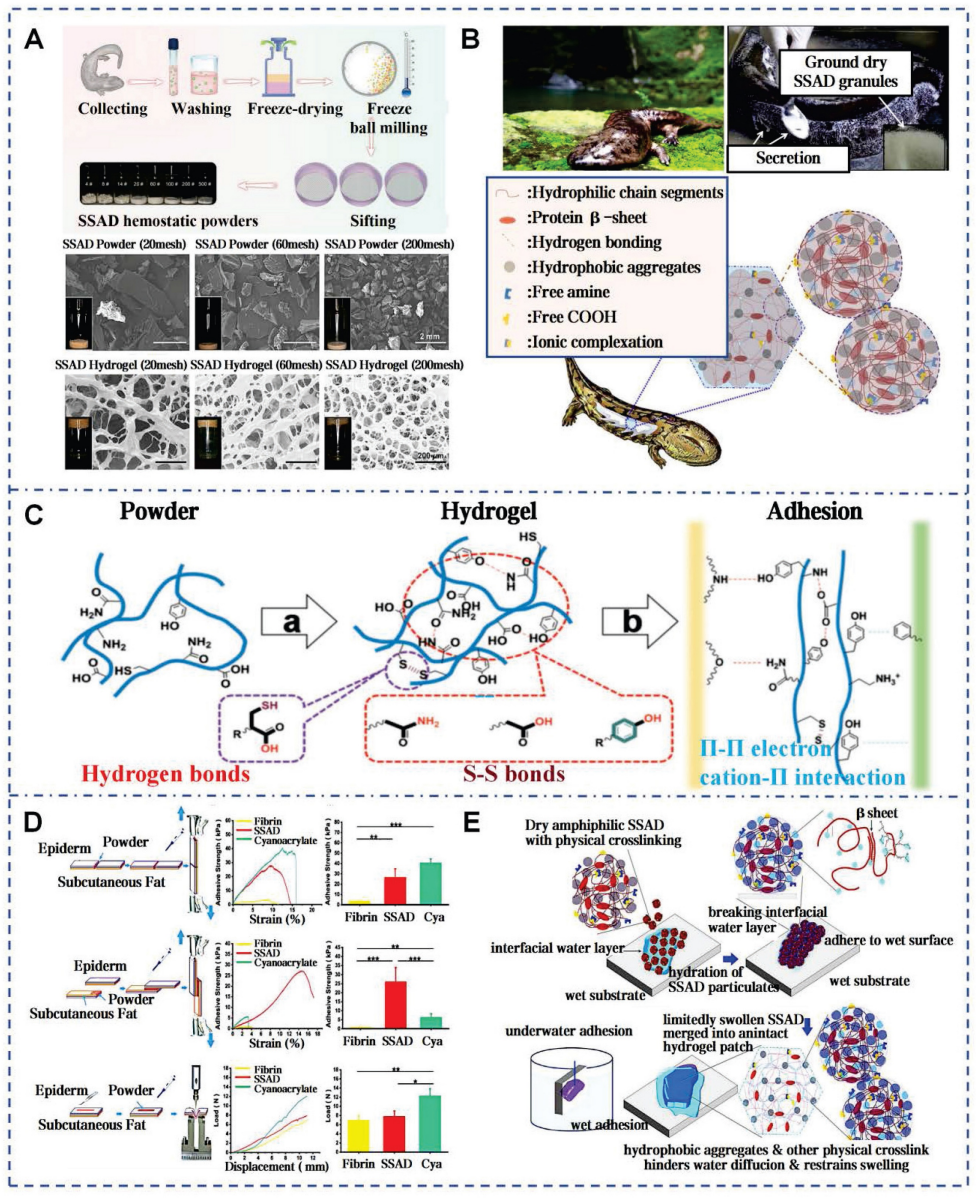
Self-assembled SSAD hydrogel: mechanism and adhesion. A) Preparation process of the SSAD powders and the porous structures of the corresponding hydrogels. Reproduced with permission 38. Copyright 2021, John Wiley and Sons. B) Self-assembled amphiphilic granular SSAD with strong wet adhesion. Reproduced with permission 86. Copyright 2022, Elsevier. C) The schematic mechanism interpretation of hydrogel formation and adhesion of SSAD 40, 86. Reproduced with permission 40. Copyright 2019, John Wiley and Sons. Reproduced with permission 86. Copyright 2022, Elsevier. D) *Ex vivo* adhesive properties of SSAD with CAs and fibrin glues. Reproduced with permission 40. Copyright 2019, John Wiley and Sons. E) Schematic illustration of dry SSAD particulates' self-assembly and adhesion mechanism in water. Reproduced with permission 86. Copyright 2022, Elsevier.

Zhang *et al.* pioneered the extraction of SSAD through freeze-drying and grinding, yielding approximately 2 g of powder per adult salamander monthly (Figure 4A) 40. SSAD exhibits rapid hydration kinetics, forming a porous structure with an average pore size of 107.08±9.1 μm, which enhances stability through progressive densification of cavity walls. The natural adhesiveness of SSAD is attributed to functional groups that facilitate bonding (Figure 4B). Phenolic hydroxyl groups and amino acids donate hydrogen bonds, enhancing biological adhesion via hydrogen bonds and van der Waals forces. Benzene rings form strong substrate interactions through π-π electronic or cation-π interactions on hydrophobic surfaces. Additionally, S-S bonds reinforce the 3D structure of the hydrogel (Figure 4C). *In vitro* tests on pig skin showed that for edge-to-edge bonding, the shear bonding strength of SSAD is 26.66±8.22 kPa, which is similar to that of CAs (40.71±3.71 kPa), and is significantly higher than the bonding strength of fibrin glue (3.76±0.16 kPa) (Figure 4D). The presence of growth factors VEGF, PDGF, EGF, HGF, and bFGF in SSAD enhances wound healing by promoting re-epithelialization, neovascularization, and stem cells recruitment via MAPK pathway activation. *In vivo* studies showed that SSAD reduced healing time and promoted scarless healing, with complete biodegradation in 3 weeks 40. Comparative analyses with *Yunnan Baiyao* revealed superior performance in both *in vitro* and *in vivo* models 38. Stability studies confirmed that SSAD exhibits instantaneous adhesion, supporting 50 g within 20 seconds and sustained this performance over 7 days (Figure 4E). This attributed to the amphiphilic protein components that eliminate hydration layers and form hydrophobic cross-links 86. The SSAD offered a multifunctional platform that integrates rapid adhesion, controlled biodegradation, and growth factor delivery.

### 3.2. Snail mucus

**Figure 5:**
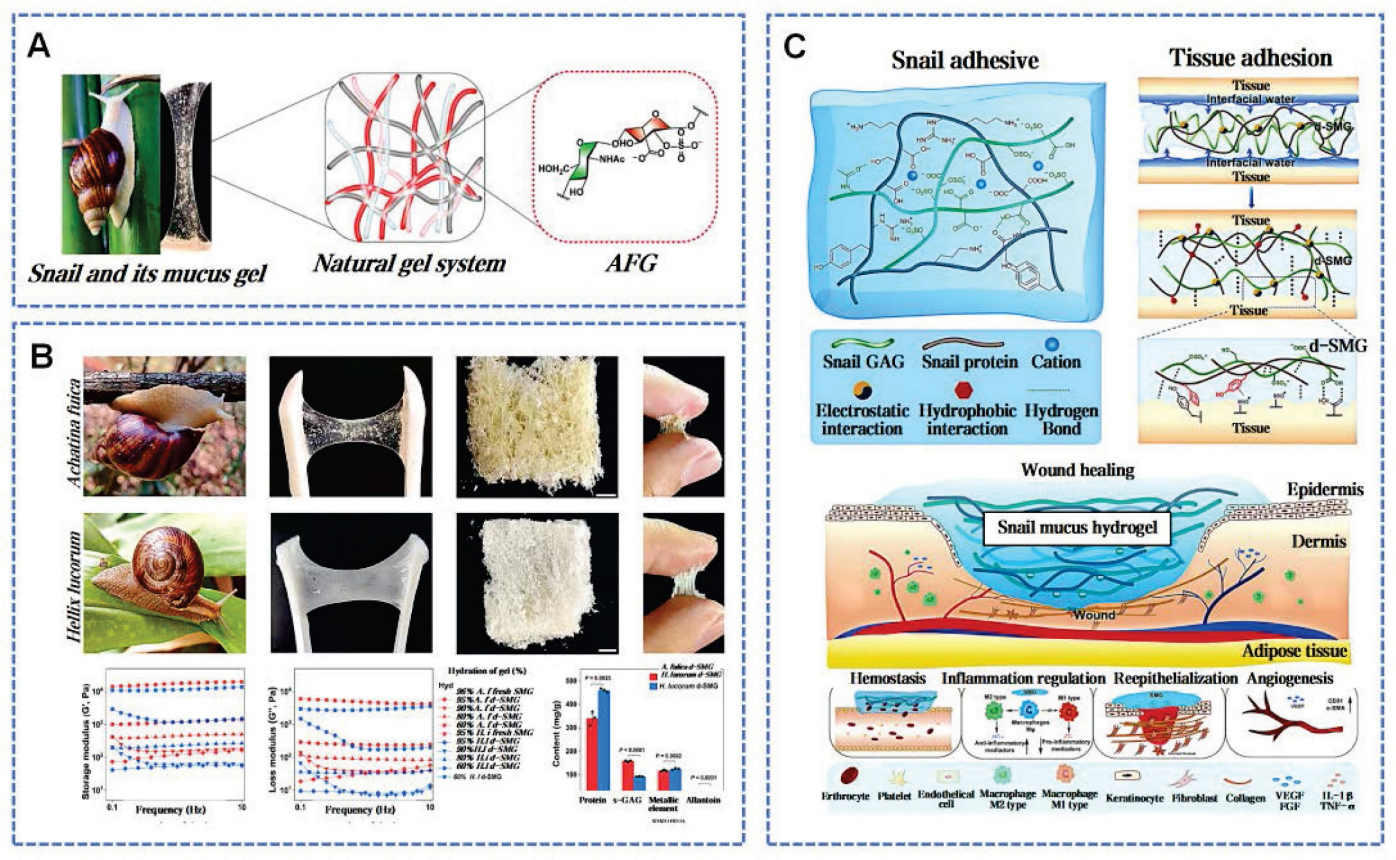
Snail mucus bioactivity and wound healing mechanism. A) Snail mucus and main bioactive glycosaminoglycan. Reproduced with permission 94. Copyright 2023, Elsevier. B) d-SMG derived from two snail species. Reproduced with permission 41. Copyright 2023, Springer Nature. C) Schematic interpretation of the mechanism d-SMG in wound healing. Reproduced with permission 41. Copyright 2023, Springer Nature.

Terrestrial gastropod snails secrete snail mucus (SNM) to preserve surface skin humidity and reduce the intake of contaminants. Snail pedal mucus exhibits robust interfacial adhesion, enabling resistance to detachment forces up to about 22 times body weight across varied substrate angles 87. Snail mucus, a wondrous byproduct of the snail's instinct for survival, has also garnered human fascination for its unique therapeutic applications. In recent years, SNM is widely known for its rich bioactive components and broad application prospects. Existing research has explored the key pharmacological properties of snail mucus: enhancing cell proliferation and migration, angiogenesis, antimicrobial activity, free radical neutralization, and tumor growth inhibition 88. Particularly, the key components of snail mucus have been a focus of research. Glycosaminoglycans (GAGs) were first extracted from the African giant snail *A. fulica* in 1996 89. GAGs not only participate directly in the construction of the ECM but also regulate the activity and inflammation-mediated functions of immune cells by intervening in cell signaling pathways (Figure 5A) 44. The protein concentration in SNM is close to 4.8 mg/mL 90. These proteins contain mucins, lectins, antimicrobial peptides, and growth factors, similar to human EGF and FGF, contributing to wound healing by reducing infection and inflammation 12. The lectins in SNM are 70 kDa glycoproteins, and about 10% of their composition is carbohydrates, primarily N-acetylglucosamine 91. Allantoin is a highly osmotic molecule in SNM, which can significantly enhance tissue absorption and retention of moisture. Ethanolamine in SNM inhibits key inflammatory enzymes by binding their active sites 88.

Assays based on cellular models revealed that SNM enhances cellular proliferation and migration, as well as increases the expression of adhesion factors in HaCaT and Hair Follicle cells 92. Wu *et al.* prepared dried snail mucin gel (d-SMG) from *Achatina fulica* and *Helix lucorum* (Figure 5B) 41. The d-SMG can rapidly form a strong adhesive on damp surfaces, suitable as natural wound adhesives. Positively charged amino or guanidino groups interact electrostatically with negatively charged sulfate and carboxyl groups in the sulfated GAGs, forming stable gel-like structures. Hydroxy, aromatic, and aliphatic amino acids in SNM facilitate extensive hydrogen bonding, π-π interactions, and hydrophobic interactions during gel formation. Divalent cations like Ca²⁺ and Mg²⁺ enhance the elasticity of the SMG through chelation and electrostatic interactions, providing structural support for adhesion. The supramolecular synergy within SMG is responsible for creating strong cohesive forces and excellent tenacity (Figure 5C) 93. Animal studies confirm d-SMG's hemostatic properties, biocompatibility, and biodegradability. SNM notably accelerates the healing in normal and diabetic rats, with superior results in granulation tissue, collagen deposition, and neovascularization compared to alginate dressings. Further analysis indicated that d-SMG could facilitate the polarization shift from pro-inflammatory M1 to anti-inflammatory M2 macrophages 41. These insights provide theoretical and material guidance for the design of bio-inspired tissue adhesives and bioengineering scaffolds.

### 3.3. Mussel mucus

**Figure 6:**
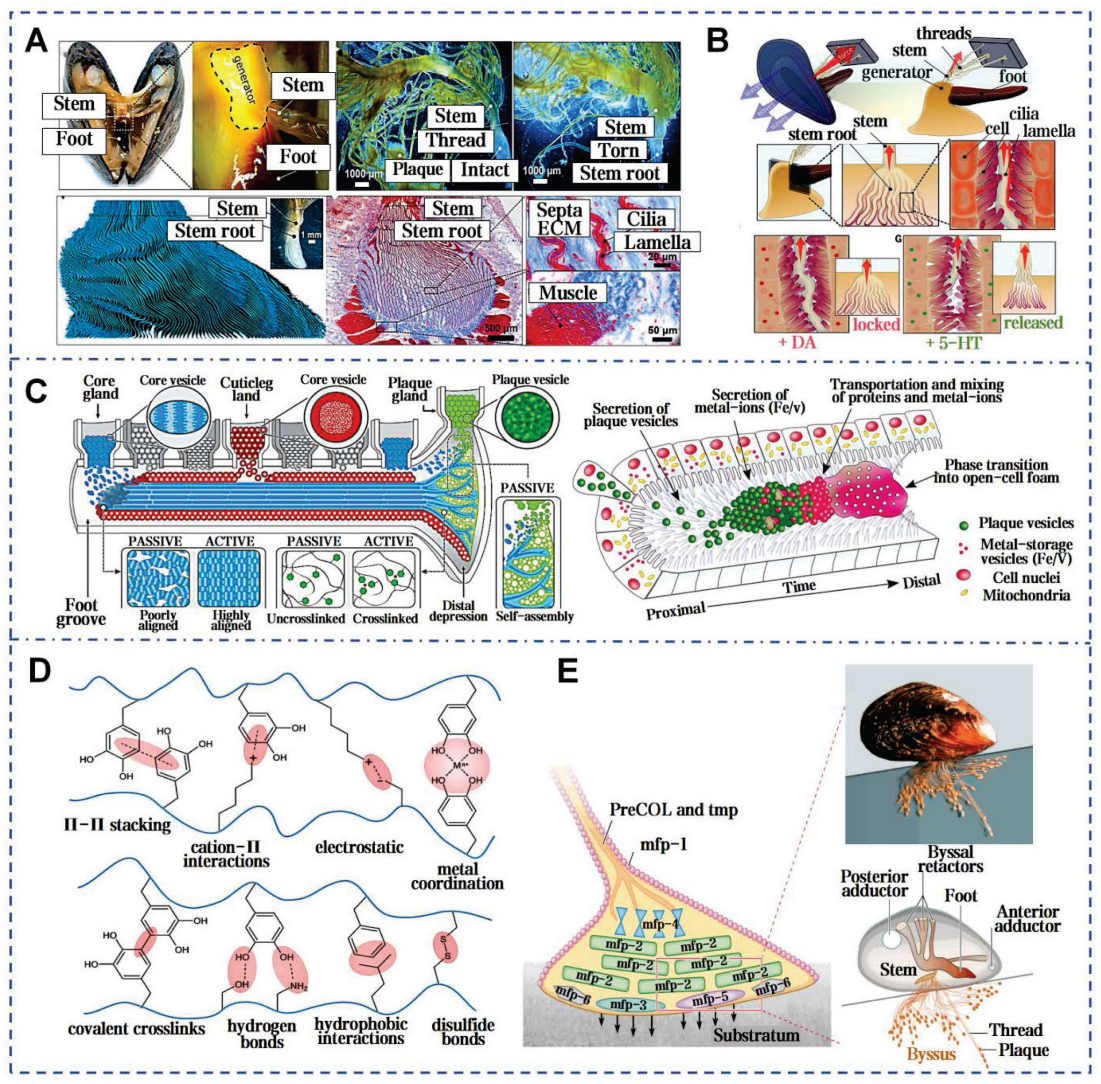
Mussel byssus attachment system: structure, function, and molecular interactions. A) The intricate structure of a mussel's byssus attachment system, including the generator region that produces the stem and its root. Reproduced with permission 100. Copyright 2023, The American Association for the Advancement of Science. B) How wave forces acting on the mussel are transmitted through the byssus into the stem and generator. Reproduced with permission 100. Copyright 2023, The American Association for the Advancement of Science. C) Schematic model of the secretion process during plaque formation 97, 105. Reproduced with permission 97. Copyright 2017, Springer Nature. Reproduced with permission 105. Copyright 2021, The American Association for the Advancement of Science. D) Overview of the proposed different adhesive and cohesive molecular interactions found in mussels. Reproduced with permission 99. Copyright 2018, John Wiley and Sons. E) The mussel byssal adhesion manifests through a dense protein framework. Mfp-1 functions as an outer cuticle for thread protection. Reproduced with permission 99. Copyright 2018, John Wiley and Sons.

Surge, storm, salinity erosion, temperature fluctuations, biofouling, and other natural factors all contribute to the complexity of the marine environment 95. However, numerous marine organisms, mussels in particular, still adhere strongly to substrates under ocean currents and maintain long-term stability. Mussels, dwelling in the intertidal zone, rank among the strongest natural adhesive sources in the wild 96. When encountering seawater, the natural mucus secreted by the mussel's foot glands rapidly solidifies into byssal threads and forms byssal plaques at the point of attachment to substrates (Figure 6A). Mussel mucus exhibits extremely strong adhesion and outstanding water resistance, solidifying rapidly to firmly attach to surfaces such as rocks, ships, glass, and corals (Figure 6B) 97. Additionally, mussel mucus also adheres strongly to inert anti-adhesive materials. In the shipbuilding industry, it can even replace conventional fastening methods like screws and rivet welding. Pujol *et al.* initially elucidated the composition and structure of mussel mucus (byssus), identifying mussel foot proteins (Mfps) and other 3 main categories proteins (Figure 6C) 98. All Mfps contain dihydroxyphenylalanine (Dopa), a hydroxylated tyrosine derivative critical for adhesion. Dopa contributes to the waterproof and versatile adhesive properties of Mfps through interactions such as hydrogen bonding, π-π interactions, cation-π interactions, metal ion chelation, and electrostatic attraction (Figure 6D) 99. The reduced catechol group adheres strongly to inorganic surfaces, but weakening upon oxidation. To counteract oxidation, mfp-6 at the adhesive plaque interface provides antioxidative protection, preserving catechol's reduced state and enhancing adhesion (Figure 6E) 100.

The adhesive properties of mussel mucus are not limited to mechanical bonding, also extend to wound healing and tissue repair. This is attributed to Mfps facilitating cell adhesion and migration 101. Furthermore, Dopa in Mfps is linked to anti-inflammatory properties, reducing the expression of pro-inflammatory cytokines. The oxidation of Dopa to Dopa quinone and its subsequent reactions form a cross-linked network, potentially strengthening the ECM for damaged tissues rebuilding 102. The catechol groups in mussel mucus are recognized for their multifunctional role in supporting coagulation, anti-inflammatory, antioxidant effects and promoting cell adhesion in a moist environment. A catechol-modified levan hydrogel demonstrated low immunogenicity, biocompatibility. This bioadhesive demonstrates an adhesive strength of up to 42.17±0.24 kPa under moist conditions, about 3 times higher than fibrin glue and accelerates the growth and migration of NIH3T3 fibroblasts and HaCaT keratinocytes 103. Lee *et al.* innovated a dopamine-based surface modification technique by oxidative self-polymerization, forming polydopamine layers of tunable thickness on various substrates 104. The detachment mechanism of mussel byssus from living tissues reveals an interface with controllable adhesion, crucial for clinical applications like implantable biomaterials and detachable biosensors.

### 3.4. Other animal mucus

Similar to snail mucus, slug mucus is also a terrestrial gastropod secretion that showed comparable efficacy in promoting wound repair. Slug mucus possesses large content of bioactive components, such as hemocyanin beta, SSD domain-containing protein, calcium-transporting ATPase and phospholipase C. These components work together to achieve a lap-shear force of approximately 1.1 N and enable rapid hemostasis in liver trauma in less than 15 seconds 106. Polysaccharides derived from natural loach mucus can significantly inhibit leukocyte migration, showing superior anti-inflammatory activity compared to the dexamethasone sodium phosphate 107. Frog skin mucus enhanced wound healing through TGF-β1 pathway activation, while radiation injury models further validate the efficacy of amphibian cutaneous mucus peptides in resolving complex tissue damage 108. Earthworm mucus extract (EE) orchestrated wound healing by stimulating cell proliferation, collagen synthesis and increasing the number of early white blood cells, neutrophilic granulocytes, and platelets 109. EE-mediated fibroblast cycle regulation specifically demonstrated mitochondrial membrane potential restoration in diabetic wounds 110. Furthermore, as a snake extract from *Bothrops atrox*, hemocoagulase can rapidly convert fibrinogen into fibrin for hemostasis and promotes wound healing through the synergistic effects of platelet activation and fibrin mesh formation. This unique rapid hemostasis property provides a foundation for its application in advanced wound dressings 111. These comparisons underscore the evolutionary conservation of bioactive mucus components, particularly in species subjected to frequent cutaneous injuries, suggesting phylogenetic patterns could guide future bioprospecting strategies for precision wound therapeutics.

## 4. Plant-Derived Natural Mucilage and Their Properties

Plant mucilage is generally biosynthesized by specialized cells or tissues, including mucilage glands, seed coat cells, or special structures on the leaf surface 112. For example, the Golgi apparatus of testa in angiosperms can secrete a special pectic complex polysaccharide, whose seeds are known as myxospermy 113. The mucilage of myxospermy is often in a dehydrated state. However, when myxospermy is exposed to water, the mucilage absorbs water and swells, breaking through the primary wall, completely wrapping around the seed's periphery and forming a gelatinous layer of mucilage on the surface of the seed 114. The plant mucilage is composed of polysaccharides, proteins, and other bioactive compounds. Plant mucilage plays multiple roles in plant physiological processes, including environmental adaptation, seed protection and dispersal, growth promotion, aiding in moisture and nutrient absorption, as well as capturing and digesting prey in carnivorous plants 115. Moreover, plant mucilage holds extensive uses in daily life and industrial applications. Carob seeds mucilage can be used as a thickener and emulsifier in food industry 116. Okra mucilage is rich in soluble dietary fibers, which can promote gastrointestinal motility, protect gastric mucosa, reduce cholesterol absorption, and facilitate lipid-lowering and laxative effects 117. Similar to flocculant, the polysaccharides present in okra mucilage can agglomerate and carry away microplastics in the water 118. This section mainly focuses on the adhesion effects, preparatory methods, and potential in promoting wound healing of natural plant mucilage derived from okra and aloe.

### 4.1. Okra mucilage

**Figure 7:**
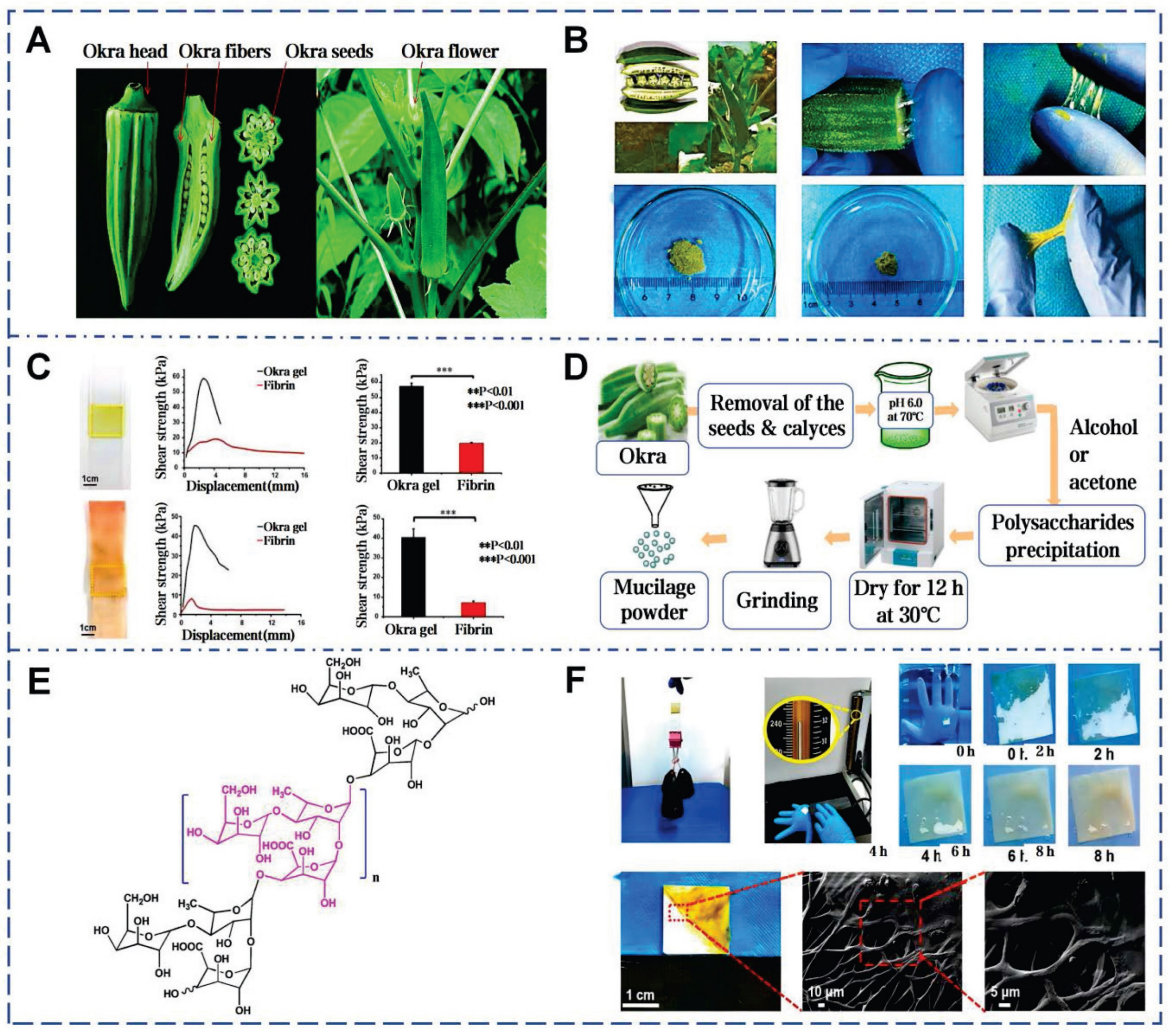
Okra mucilage: structural, chemical, and mechanical evaluations. A) Picture of okra plant and okra internal structure. Reproduced with permission 126. Copyright 2021, Springer Nature. B) Okra mucilage and mucilage freeze-dried powder. Reproduced with permission 55. Copyright 2022, John Wiley and Sons. C) Comparison of adhesion between okra mucilage and fibrin glue on glass and pig skin. Reproduced with permission 55. Copyright 2022, John Wiley and Sons. D) Extraction and isolation of mucilage from okra pods. Reproduced with permission 118. Copyright 2020, Elsevier. E) Chemical structure of okra mucilage polysaccharide. Reproduced with permission 118. Copyright 2020, Elsevier. F) Load-bearing test, underwater adhesion test and SEM images of okra mucilage. Reproduced with permission 55. Copyright 2022, John Wiley and Sons.

Okra is an herbaceous annual plant belonging to the genus Abelmoschus in the Malvaceae family (Figure 7A). Conrad *et al.* first isolated mucilage from okra pods, beginning the identification of its acidic polysaccharides (Figure 7B) 119. Subsequent research established a direct correlation between the polysaccharide content and the viscosity of okra mucilage (Figure 7C) 120. Okra mucilage is primarily composed of pectin, polysaccharides, and glycoproteins, with pectin being the major constituent 54. Galacturonic acid, galactose, rhamnose, glucose and other monosaccharides are interconnected, forming a robust structure. High content of galactose and galacturonic acid indicate that okra polysaccharides (OPS) possess characteristics akin to pectin, supported by the RG-I region 121. OPS can be used as an adhesive in Naproxen sodium tablets, surpassing traditional starch in adhesive strength 122. In addition, different extraction conditions can also affect the molecular structure within okra mucilage, thereby impacting its rheological properties and adhesive effectiveness (Figure 7D) 123.

Glycosidic bonds and ionic groups on okra polysaccharide chains can form intermolecular and intramolecular hydrogen bond networks, causing chain expansion and increasing viscosity (Figure 7E) 124. These high molecular weight polysaccharides offer abundant interaction sites to form a robust 3D network, which is beneficial for wound coverage and protection (Figure 7F) 125. According to traditional medicinal practices, the mucilage derived from pounded okra fruits can be used directly to heal skin wounds and subcutaneous abscesses 126. When powder of freeze-dried okra mucilage rehydrated, it forms a highly viscous natural okra hydrogel (OHG) 55. OHG can substantially reduce the levels of TGF-β and IL-1β in the damaged tissue, and enhances collagen deposition and tissue maturation. The adhesive strength of OHG (glass: 57.6±1.9 kPa, pigskin: 40.6±4.3 kPa) is about 3 times higher than that of fibrin glue on glass substrates and about 6 times higher on porcine skin. Compared with chitosan hemostatic agents, OHG has a shorter coagulation time. Notably, this work firstly elucidates the potential of okra mucilage as an innovative natural biomaterial in stimulating platelet polarization and promoting tissue regeneration.

### 4.2. Aloe mucilage

**Figure 8:**
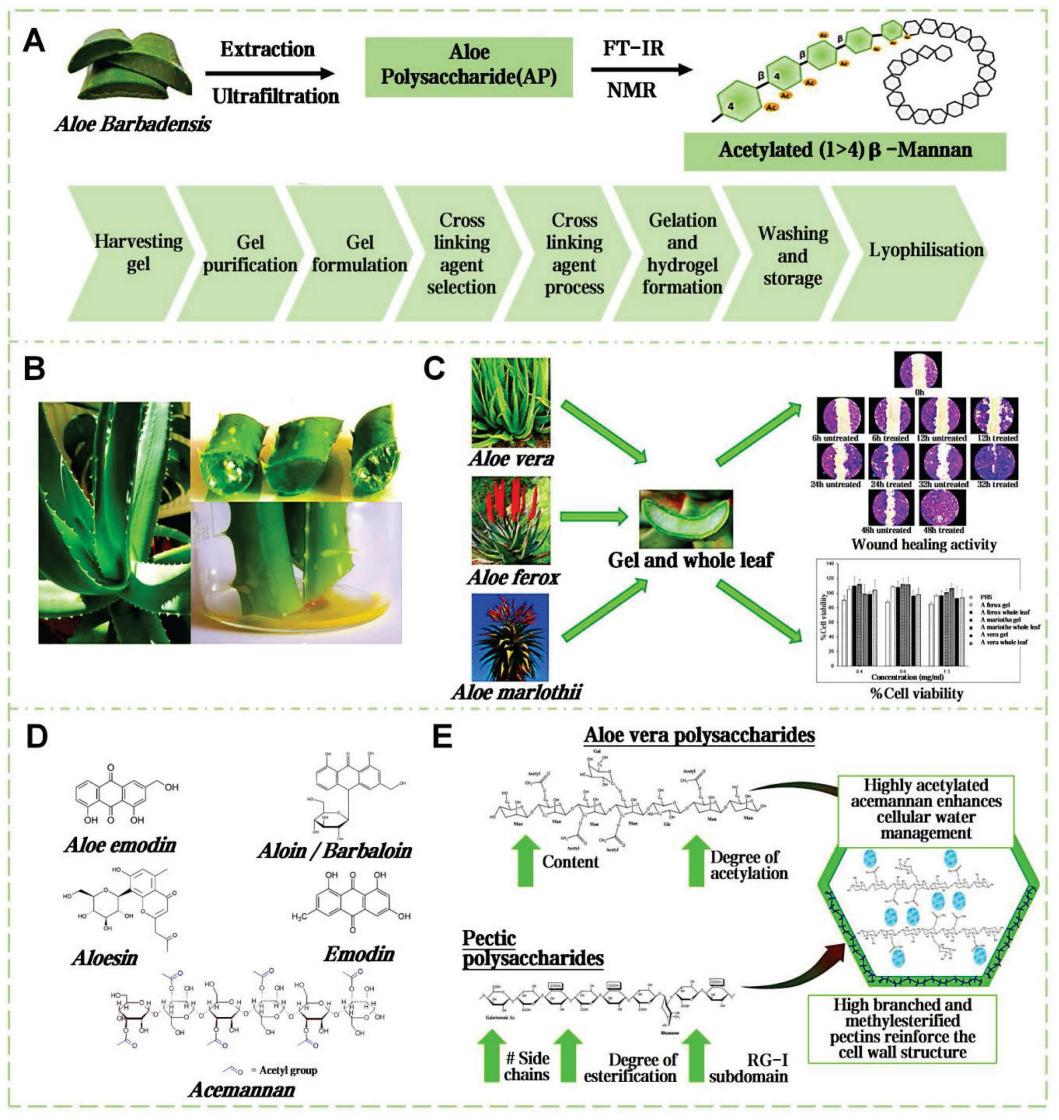
Comprehensive analysis of aloe mucilage: from extraction to biological properties. A) Extraction and isolation routes of natural aloe mucilage. Reproduced with permission 142. Copyright 2020, Elsevier. B) Picture of aloe plant and aloe internal structure. Reproduced with permission 134. Copyright 2023, MDPI. C) Three species of aloe mucilages for cell growth and wound healing promotion. Reproduced with permission 143. Copyright 2017, Elsevier. D) The main chemical components of aloe vera mucilage. Reproduced with permission 134. Copyright 2023, MDPI. E) The molecular mechanism of moisture retention properties of aloe mucilage. Reproduced with permission 131. Copyright 2024, Elsevier.

Aloe vera, a perennial herb of the Liliaceae family, has globally medicinal applications for over 23 centuries 127. The structure of the aloe leaf is tri-layered. Outermost layer is fibrous epidermis, preventing excessive moisture evaporation and external environmental damage (Figure 8A). The outer leaf area secretes a yellowish latex. The inner leaf's thin-walled tubular cells secrete a colorless, tasteless mucilage, known as aloe gel (Figure 8B) 128. Dry weight analysis shows the key components of aloe gel are polysaccharides, followed sequentially by minerals, proteins, lipids, and phenolic compounds 129. The acetylated mannans and polymannoses can establish hydrogen and ionic bonding with polar entities on the surface encountered. The carboxyl groups can ionically bond with skin cations, the hydroxyl groups of phenolic compounds can form covalent bonds with thiol groups in skin proteins, further stabilizing the adhesive interface 130. Concurrently, the moisture in aloe gel aids in the relative movement and reorientation of polysaccharide chains, thus improving the adhesion effect 131. Gao *et al.* delineated the pharmacological activity and clinical utilities of aloe gel, including its roles in tissue repair, radiation recovery, burn healing, acne, antioxidation, antiviral, antibacterial, anti-inflammatory, antidiabetic, anticancer, skin protection and immunity boosting (Figure 8C) 132.

Aloe gel enhances wound healing via synergistic mechanisms (Figure 8D) 133. Acemannan polysaccharides activate macrophage phagocytosis and stimulate TGF-β-mediated collagen synthesis. Interlinked polysaccharide chains create a 3D framework, which can augment the oxygen concentration and microcirculation within the wound vicinity (Figure 8E). Lupeol, saponins, salicylic acid, urea nitrogen, cinnamic acid, dihydroxyanthraquinone, anthraquinone derivatives, as well as those containing phenolic and sulfur elements, together form a complex system with antibacterial and anti-inflammatory properties 134. The experiment results show that aloe mucilage at a 40% (w/v) concentration was effective in inhibiting the growth of gram-negative bacteria 135. Moreover, essential amino acids provide the necessary substrate for tissue repair and cell regeneration. Vitamins, organic acids, minerals, and other various trace elements are crucial in shielding wounds from oxidative stress. Lectins, gibberellins, and growth factors can fortify the body's innate reparative capabilities 136. Aloe mucilage can enhance fibroblast and blood vessel counts in burn wound healing 137. In addition, aloe mucilage has demonstrated efficacy in remedying insect stings and ringworm, also applied to treat various viral skin lesions 138. Blending freeze-dried aloe mucilage powder with varying quantities of water, which can serve as a coating for bamboo fiber sutures 139. This composite dressing can be customized to the size and shape of the wound. Pan *et al.* mixed an aloe hydrogel matrix with aloe-derived exosome nanoparticles to create a new wound dressing (ADENHs) 140. ADENHs treatment can significantly reduce serum IgE levels in atopic dermatitis model, decrease the expression of inflammatory cytokines in diabetic wound. Furthermore, the integration of aloe mucilage into the E-skin architecture can be used as an advanced wound dressing, that can monitor and respond to the healing process 141.

### 4.3. Other botanical mucilages

There are also many unexplored natural plant mucilages with potential to promote wound healing. Yam mucilage facilitates effective hemostatic management in complex tissues and organs with its blood-activated gelation and robust hemostatic adhesion capabilities 144. The glial mucus in Tunisian cactus has been shown to have antibacterial and antifungal properties 145. Mustard mucilage contains glucosinolates, which possesses antimicrobial properties 146. The bark of the peach tree can secrete a type of natural plant mucilage, known as peach gum 147. Peach gum is rich in various polysaccharides and phenolic compounds, which endow it with certain anti-inflammatory and antioxidant properties. Currently, peach gum has been successfully applied in the preparation of adhesives and hydrogel materials. Oliveira *et al.* summarized the application of mucilage from flaxseed, Brazilian cactus pear, and chia seeds in wound treatment 133. They found that these plant-derived mucilages are rich in bioactive components, such as Omega-3 fatty acids and vitamin E, which show potential in accelerating wound healing and reducing infection risk.

## 5. Complex-Sourced Natural Mucus and Their Properties

The ability to secrete mucus with distinctive properties is not confined to just fauna and flora. In fact, the kingdom of biological mucus pervades every species in nature, demonstrating remarkable diversity in morphology and function. Complex-sourced natural mucus is not a single substance, but a general term for a class of dynamic biohydrogels. Depending on their origin and function, they are often referred to by various terms such as "slime, hydrogel, biofilm, mucilage, glycocalyx exopolysaccharides and extracellular polymeric substances (EPS)". Complex-sourced natural mucus can also be found in ecological contexts, such as the EPS of biological soil crusts or the organic aggregates of marine snow 8. These mucous substances present great application potential in biomedicine and material science. This section focuses on two typical types of mucus with natural biopolymer characteristics: Propolis (a complex substance derived from both animal and plant components) and microbial EPS (exemplified by BC, a structurally defined exopolysaccharide for clinical application). We discussed the biological origins, chemical compositions, and properties and their prospective applications in enhancing wound healing. By deeply exploring the properties and functions of these unconventional bioadhesives, we can discover more natural resources, providing innovative ideas and methodologies for the advancement of biomaterials treatment methods.

### 5.1. Propolis

**Figure 9:**
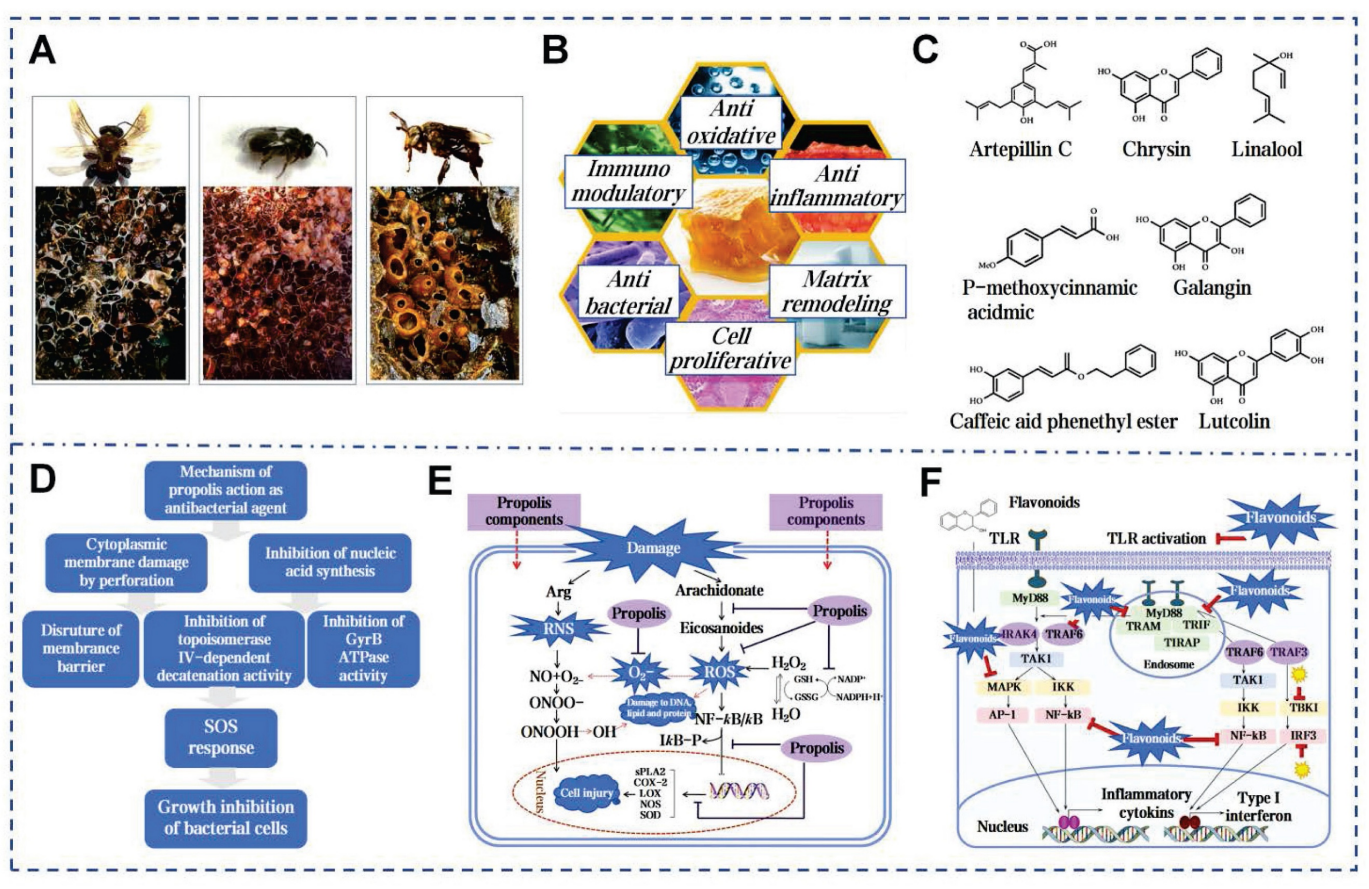
Multifaceted biological activities and molecular mechanisms of propolis. A) The images of stingless bees and their propolis. Reproduced with permission 153. Copyright 2020, Elsevier. B) The main mechanisms of propolis in promoting wound healing. Reproduced with permission 72. Copyright 2015, Oxford University Press. C) Main functional compounds of propolis. Reproduced with permission 63. Copyright 2022, John Wiley and Sons. D) Mechanism of propolis action as anti-bacterial agent Reproduced with permission 73. Copyright 2018, Elsevier. E) The molecular mechanism of the propolis-mediated protective effect during the oxidative stress Reproduced with permission 73. Copyright 2018, Elsevier. F) Antibacterial mechanism of flavonoids in propolis Reproduced with permission 73. Copyright 2018, Elsevier.

Propolis, a fragrant resinous substance, is synthesized by worker bees that mixing plant exudates such as buds, leaves, and sap from tree wounds with secretions from their own glands (Figure 9A). The medicinal value of propolis has been documented in ancient texts worldwide and dates back 3,000 years (Figure 9B) 148. Bees utilize propolis to fortify their hives against environmental hazards and intruders, or to encase carcasses and prevent decay and microbial growth. The adhesiveness of propolis arises from its complex chemical composition, primarily consisting of resin, beeswax, essential oils and pollen 149. Resin acids and gum form hydrogen and ionic bonds. Flavonoids enhance adhesion to nonpolar surfaces and terpenes promote π-π stacking. Beeswax components contribute through hydrophobic and Van der Waals forces. Additionally, enzymes secreted by bees during processing catalyze cross-linking reactions, further strengthening the network and enhancing propolis's mechanical properties and adhesion on various surfaces (Figure 9D) 150.

Propolis contains over 600 compounds, with coffee acid phenethyl ester (CAPE) being the most studied 151. CAPE can reduce histamine release and inflammatory cytokine production, while also acting as a potent inhibitor of the NF-κB pathway 152. Emodin and Kaempferol are key antiallergic components 153. The ethanol extract of propolis shows stronger antioxidant activity than vitamin C and E (Figure 9E) 154. Propolis has been confirmed to inhibit various bacteria. The active components of propolis can attach to the bacterial cytoplasmic membrane, leading to membrane perforation. Its flavonoid compounds inhibit the activity of topoisomerase IV, suppressing bacterial growth (Figure 9F) 155. Propolis contains over 12 mineral elements. The Zn^2+^ can aid skin follicle regeneration and suppress bacterial growth 155. Martinotti *et al.* outlined the role of propolis in stimulating wound matrix remodeling and increasing the components of the ECM in the early stages of wound repair 72. Studies indicate that Brazilian red propolis enhances wound healing by reducing neutrophils and macrophages at the wound site 156. The ethanol extract of Chinese propolis reduced the buildup of reactive oxygen in fibroblasts by modulating antioxidants gene expression 157. Propolis nanoparticles (PNPs) are synthesized from propolis extract using a pH differential method. PNPs significantly enhance the levels of antioxidant enzymes SOD and glutathione in wound tissue and upregulate TGF-β. These effects indicate their potential in clinical skin wound treatment by promoting collagen formation and angiogenesis 71.

### 5.2. Microbial EPS

**Figure 10:**
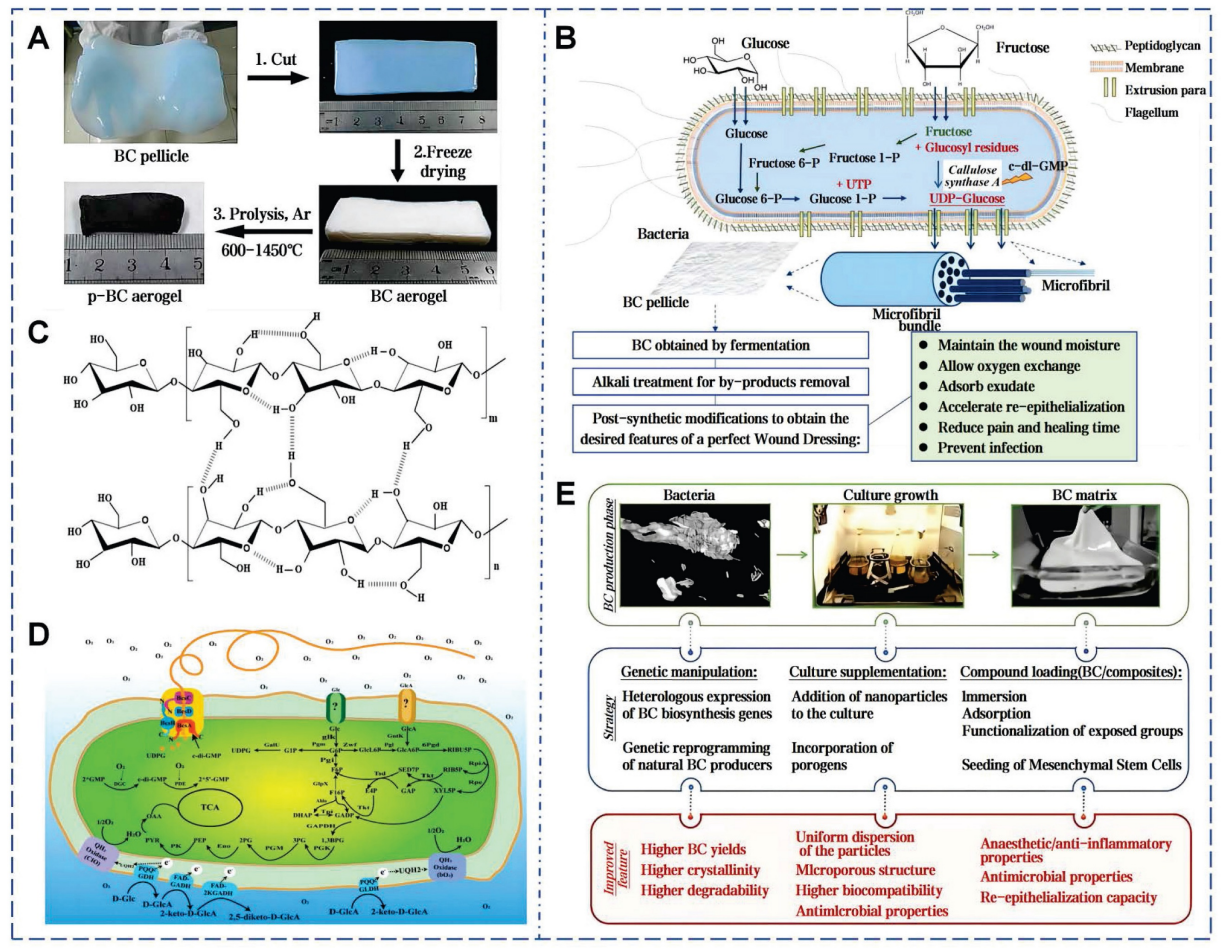
Comprehensive fabrication and properties of BC-based wound dressings. A) Various steps involved in the fabrication of BC-based materials. Reproduced with permission 169. Copyright 2021, John Wiley and Sons. B) The steps involved in the production of a BC-based wound dressing. Reproduced with permission 75. Copyright 2019, John Wiley and Sons. C) Chemical structure of BC. Reproduced with permission 74. Copyright 2021, Elsevier. D) Metabolic diagram of BC produced by Acetobacter. Reproduced with permission 74. Copyright 2021, Elsevier. E) Summary of the processes involved in BC production. Reproduced with permission 75. Copyright 2019, John Wiley and Sons.

As the most primitive and proficient mucus producers in nature, microorganisms lack a unified nomenclature for their secreted mucus. Due to their unique growth characteristics, microorganisms can bind self-secreted extracellular polysaccharides with water, sediments, metabolites, and other matrices in the surroundings to form specialized EPS 158. BC is a natural polysaccharide hydrogel generated through metabolic fermentation using sugars as the main carbon source (Figure 10A) 159. In 1886, Brown first isolated BC from acetic acid fermentation tanks, and this bacterium was later named *Komagataeibacter xylinus* 160. This bacterium links β-D-glucopyranose units via β-1,4-glycosidic bonds to form nanoscale glucose polymers (Figure 10B). During secretion, the bacteria move randomly in the culture medium, resulting in an ultra-delicate 3D porous network structure. (Figure 10C) 75. The nanoscale thickness fiber network of BC provides high-density hydrophilic groups and an expanded internal surface area, enabling hydrogen bonding with water molecules and enhancing adhesion to moist surfaces 161. Compared to plant cellulose, BC exhibits greater mechanical strength, with high degrees of polymerization and crystallinity 162.

Studies have shown that EPS derived from bacteria (*Bacillus subtilis*) and microalgae (*Chlorella zofingiensis*) can accelerate wound healing. This microalgae-probiotics biogenic dressing establishes a 3D harmonized microbial community at wound sites, delivering dissolved oxygen while suppressing pathogenic colonization and modulating healing dynamics 163. BC exhibits flexibility, high purity, non-toxicity, non-irritation to skin, high biocompatibility, degradability, and renewability, making it an ideal candidate for natural wound dressings (Figure 10D) 164. Over the last century, BC-based commercial wound dressings have been marketed. They showed excellent wound adhesion, healing acceleration, and infection risk reduction in over 300 clinical trials for skin damage and burns 165. To date, various BC products are widely available. The ultrafine fibers of BC can granulation tissue adhesion, avoiding epithelium stripping during dressing changes and facilitating wound monitoring (Figure 10E) 74. The unique structure of BC can continuously absorb exudate, maintaining optimal moisture and inflammatory levels at the wound site 166. In addition, the chemical formula of BC is (C_6_H_10_O_5_)_n_, with each glucose ring featuring hydroxyl groups. These hydroxyl groups in BC molecular chains can be readily modified by functional groups such as aldehydes, carboxylic acids, and amines, resulting in different properties 167. The allylation modification to BC promoted its water absorption capacity after drying 168. Hollow BC microspheres, fabricated using microfluidic technology, can be served as innovative injectable porous scaffolds in 3D cell culture and tissue regeneration. After 48 h of culture, cells in the hollow BC microsphere scaffold proliferated to 95 µm depth, versus 10 µm in the bulk BC scaffold (within 100 µm framework) 77.

## 6. Pre-Clinical Studies for Natural Mucus in Diverse Wound Models

Wound is defined as injury to the structure of tissues or organs, customarily divided into acute and chronic wounds. Uncontrolled bleeding from acute wound is the second leading cause of pre-hospital deaths, and about 50% combat-related deaths linked to acute wounds 170. Chronic wounds could not revert to normal anatomical and functional states via the organism's innate reparative mechanisms. 1%-2% of the global population could experience chronic wounds during their lifetime 171. Given the limitations of self-healing capabilities, medical intervention is often required to facilitate treatment. In 1962, Dr. Winter found that the healing rate in a moist milieu is over twice as quick as in a dry setting, hence mitigating scar formation. This revelation prompted the FDA to establish the protocol of moist wound as a standard approach in wound management 172. Based on this concept, a lot of natural mucus has been frequently applied in wound healing. This chapter summarizes the characteristics of various wound models and their clinical treatment challenges, further analyzes the therapeutic effects of natural mucus in these models.

### 6.1. Acute skin traumas

The integumentary system acts as the first line of defense against external stimuli and injuries. Skin is one of the largest organs of the human body, with the surface area reaching 1.5 to 2.0 m^2^ in adults and about 0.21 m^2^ for newborns 173. Acute skin traumas can rely on the skin's self-repair capabilities to fully heal within 2 to 4 weeks. However, due to factors such as metabolic diseases, impaired wound microcirculation, or microbial infections, common skin injuries may develop into chronic wounds. Therefore, promoting the speed of wound healing is crucial for acute skin traumas 174.

**Figure 11:**
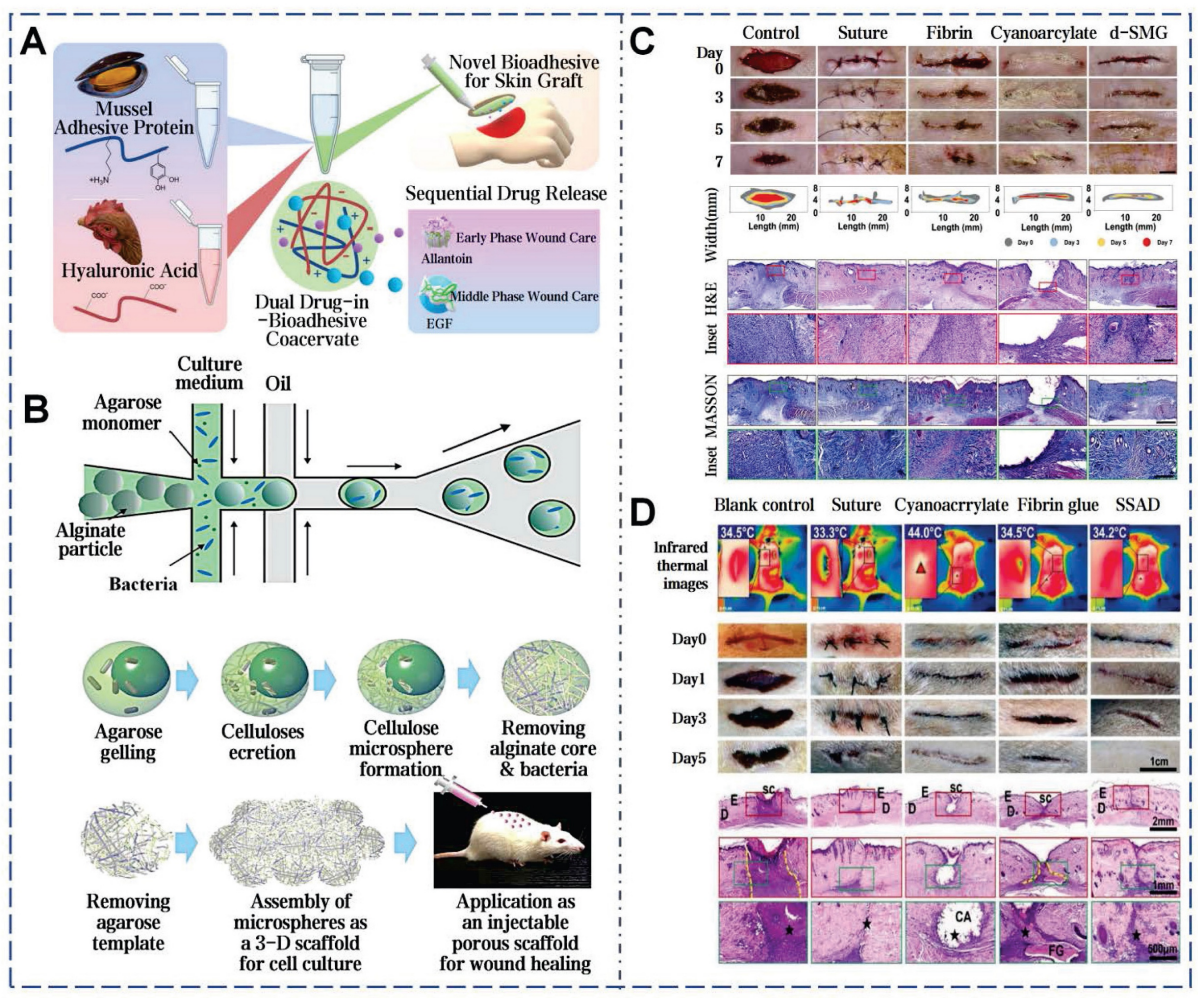
Selected cases of natural mucus utilization in routine skin wound healing. A). MAP-derived bioadhesive coacervates are utilized to facilitate wound healing following full-thickness skin transplants. Reproduced with permission 49. Copyright 2022, Elsevier. B). BC microspheres promote wound healing. Reproduced with permission 77. Copyright 2016, John Wiley and Sons. C). *In vivo* adhesion and healing effects of SNM. Reproduced with permission 41. Copyright 2023, Springer Nature. D). *In vivo* adhesion and healing effects of SSAD. Reproduced with permission 40. Copyright 2019, John Wiley and Sons.

Adhesives developed from mussel adhesive proteins still exhibit high adhesion strength under wet conditions. The dopamine residues in these adhesives can interact with other molecules and accommodate the dynamic progression of wounds, thereby enhancing wound healing following full-thickness skin transplants (Figure 11A) 175. Additionally, BC without any modification is also demonstrated effective promotion of wound healing. Compared with traditional BC membranes and solid BC microsphere scaffolds, cells on the scaffold assembled by hollow BC microspheres show a deeper penetration depth and higher proliferation rate (Figure 11B) 77. Parallel to previous findings, snail mucus is known for having unique bioactive properties and enhancing wound healing 41. After treatment with snail mucus, the epidermis is completely regenerated, with distinct hair follicles and sebaceous glands near the incision (Figure 11C). The efficacy of SSAD in wound management is also demonstrated. Zhang *et al.* compared SSAD to traditional suture, α-cyanoacrylate, and fibrin glue 40. Application of 5 mg of SSAD powder to a 2-cm full-thickness skin incision can achieve rapid hemostasis and wound closure within 30 seconds. Furthermore, SSAD modulated acute inflammatory cell recruitment to the wound site and promoted continuous basal membrane integration which resolves completely within 21 days to minimize scarring (Figure 11D). SSAD can inhibit excessive TGF-β1 and TGFB1 secretion, while enhancing FGF2-mediated intercellular signaling. This scarless regeneration was further validated by near-absent expression of EN-1 (a fibrosis-associated fibroblast marker) in SSAD-treated wounds. Transcriptomic analyses revealed upregulated genes for ECM remodeling and downregulated fibrosis-related pathways. The scar ratio in the SSAD group was only 18.08%±6.64% significantly lower than 63.87%±6.46% in the blank control group 176.

### 6.2. Diabetic wounds

Diabetic wounds are among the most challenging diseases globally, with approximately 600 million people worldwide affected by diabetes. At present, over 80 million people with diabetes worldwide are struggling with diabetic wounds and diabetic foot ulcers, resulting in amputation rates as high as 24% 177. Regrettably, the 5-year survival rate of patients who undergo amputations due to diabetes is lower than that of most cancer patients, and the treatment costs associated with diabetes-related amputations often exceed those of common cancers 178. The main etiologies of chronic diabetic wounds include difficulties in vascular reconstruction, peripheral neuropathy, and continuous activation of inflammation. Elevated blood glucose levels lead to excessive generation of ROS in HUVECs, activating pathways like protein kinase C, which induces cell damage and ultimately delaying wound healing 179.

**Figure 12:**
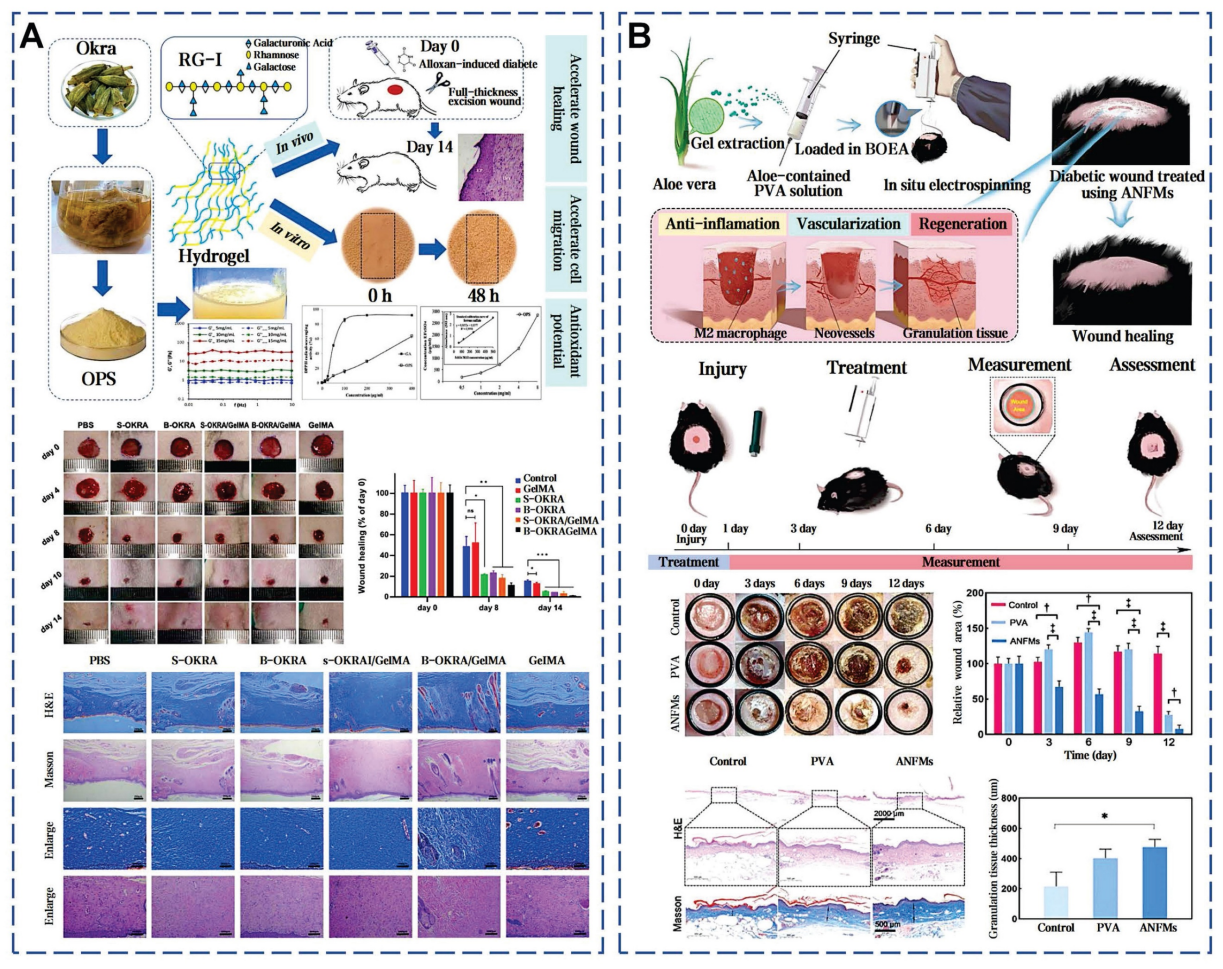
Selected cases of natural mucus utilization in diabetic wound healing. A). Okra mucilage-loaded gel promotes diabetic wound healing 52, 54. Reproduced with permission 52. Copyright 2023, Elsevier. Reproduced with permission 54. Copyright 2023, Elsevier. B). Aloe vera mucilage-derived antimicrobial nanofiber mats to promote chronic wound healing. Reproduced with permission 58. Copyright 2023, Elsevier.

Wu *et al.* delved into the application of snail mucus for diabetic wounds, demonstrating its ability to increase granulation tissue thickness and collagen deposition, as well as promote angiogenesis and epithelialization 41. The presence of GAG in d-SMG is associated with the promotion of M2 macrophage polarization through STAT3 phosphorylation upregulation. SSAD also has the significant potential in the treatment of diabetic wounds by stimulating angiogenesis and reduces inflammation 40. Notably, full-skin diabetic defect treated with SSAD showed minimal scarring. In addition, okra mucilage has demonstrated the ability to markedly enhance the healing process in diabetic wounds (Figure 12A) 52. These effects are attributed to the continuous release of various bioactive components in okra mucilage, such as okra polysaccharides, flavonoids, phenolic acids, and small molecular weight nutrients. Aloe mucilage was fabricated into aloe nanofiber membranes (ANFMs) to promote chronic wound healing. ANFMs promoted granulation tissue thickening and neovascularization, and the increase in the proportion of Ki-67 positive cells further confirms the potential to accelerate cell proliferation (Figure 12B) 58.

### 6.3. Burn injuries

Over 11 million burn cases are reported globally annually, with 180,000 fatalities attributed to burn-related complications 180. Burn injuries represent a special kind of skin injury. First-degree burns are similar to common skin injury and typically heal within one week. Second and third-degree burns extend into the dermis and the full thickness of the skin, resulting in significant immunological and barrier dysfunction 181. Infection remains a leading cause of mortality, with systemic infections occurring in 17.73% of burn patients and rising to 39.96% in third-degree cases. Moreover, the total body surface area is also employed to assess the severity of burns. Burn wound healing process represents the body's protective and adaptive responses to burn-damage tissue. During this process, macrophages stimulate fibroblasts to proliferate and secrete collagen and other ECM components, forming granulation tissue and supporting epithelial coverage 182.

**Figure 13:**
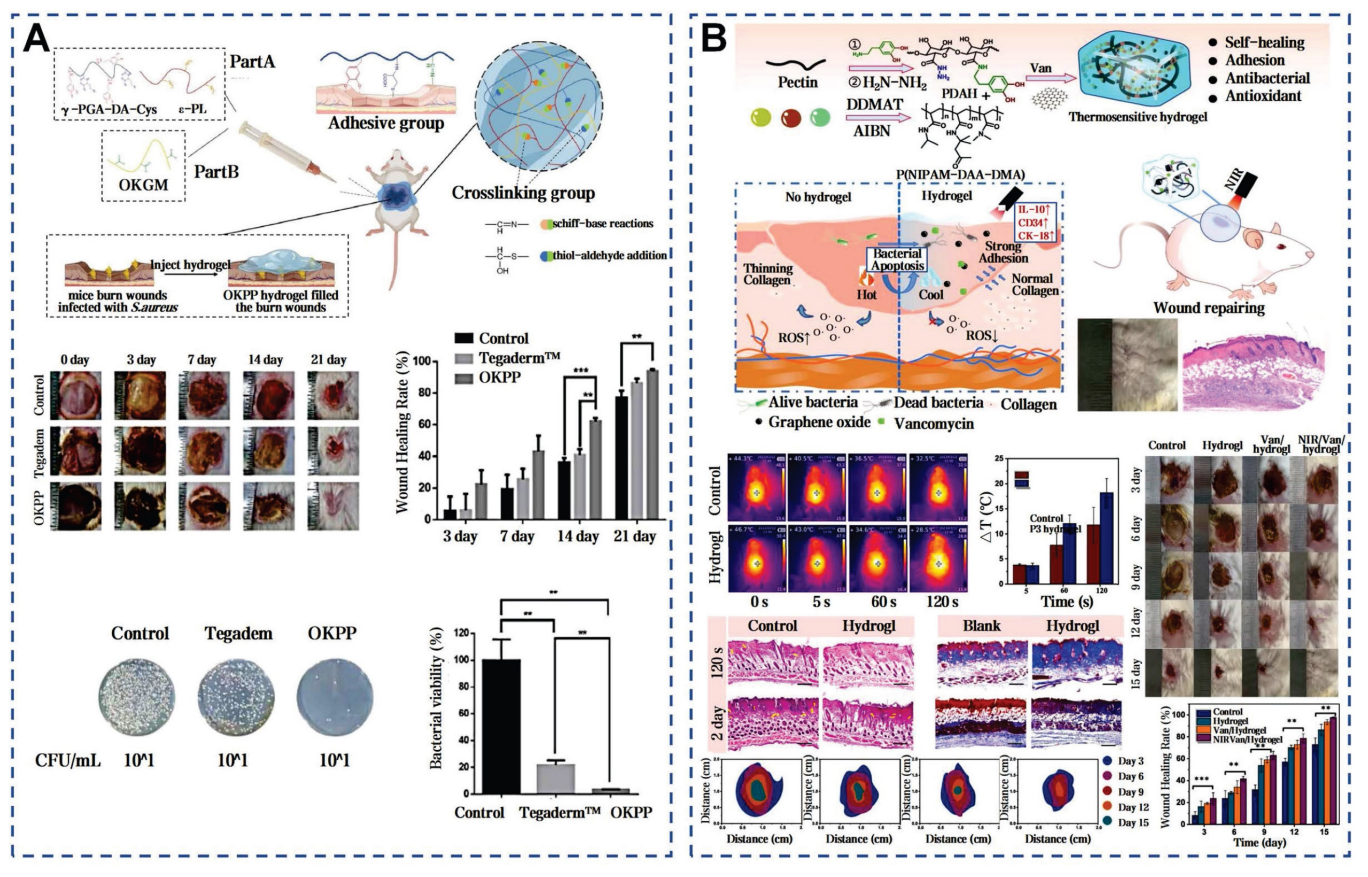
Selected cases of natural mucus utilization in burn wound healing. A) Mussel-inspired dopamine-mediated adhesive and antioxidant hydrogel for advanced burn wound healing. Reproduced with permission 46. Copyright 2022, Springer Nature. B) Mussel-inspired catechol-functionalized pectin hydrogel: a NIR-enhanced thermo-responsive composite for accelerated burn wound healing. Reproduced with permission 47. Copyright 2024, Elsevier.

Aloe vera mucilage has shown significant application prospect in burn wound healing. In patients with first or second degree burns, the healing success rate treated with aloe vera mucilage reached 95%, outperforming the sulfadiazine cream group and the framycetin cream group 60. Gauze soaked in aloe vera mucilage was more effective than petroleum jelly gauze in partial-thickness burns, with minor side effects like irritation or itching. Recent research further supported that aloe mucilage mixtures can enhance cell proliferation and migration via AKT and ERK pathway phosphorylation 183. The mussel mucus contains a high concentration of PUFAs, vitamins E and D, and omega-3 fatty acids. These lipids can significantly shorten the healing time of burn wounds 184. Due to low yield and high costs of natural mussel mucus, a range of synthetic mussel-inspired dressings have been developed 185. Chemical cross-linking can mimic the wet adhesion and self-healing abilities of mussel mucus. The mussel-inspired hydrogel reduced the healing time from 20-22 days to 12-16 days, better than the 3M Tegaderm commercial dressing (Figure 13A) 46. Another approach used dopamine functionalization to enhance antibacterial properties under near-infrared (NIR) irradiation, achieving a 97.8±0.5% wound closure rate in 15 days (Figure 13B) 47. The modified mussel-inspired mucus can be designed to enhance specific functionalities, such as antimicrobial effects, making it a promising candidate for clinical burn treatment.

### 6.4. Infected wounds

Bacterial infection is an inevitable issue during the wound healing process. Up to now, infected wounds remain the most challenging and costly wound problems globally, with severe manifestations potentially culminating in sepsis, osteomyelitis, or amputation 186. Invasive bacteria secrete various polymers to form a protective biofilm, thereby eluding the host's immune defenses and resisting antibiotic therapies. The exudates and necrotic tissues further impede the deep infiltration of antimicrobial medications. Infected wounds are accompanied by sustained, low-level inflammation and excess inflammatory cytokines release. This traps the wound healing process in the inflammatory phase, impeding the transition to the proliferation and remodeling. Even if the bacteria at the trauma site are killed by external intervention, the remaining dead bacteria and toxins in the wound area can still hinder the healing process of the wound 187. Therapeutic interventions for bacterial wound infections are generally classified into two main parts: antimicrobial therapy and facilitation of wound healing. Antimicrobial strategies now include alternatives to conventional antibiotics 188. The overuse of antibiotics has accelerated the increase in bacterial drug resistance. Additionally, metallic antimicrobial agents are frequently associated with latent toxicity concerns, environmental contamination risks, and discoloration 189. Consequently, the development of biocompatible and environmentally friendly natural mucus is expected to offer a novel solution to this problem.

**Figure 14:**
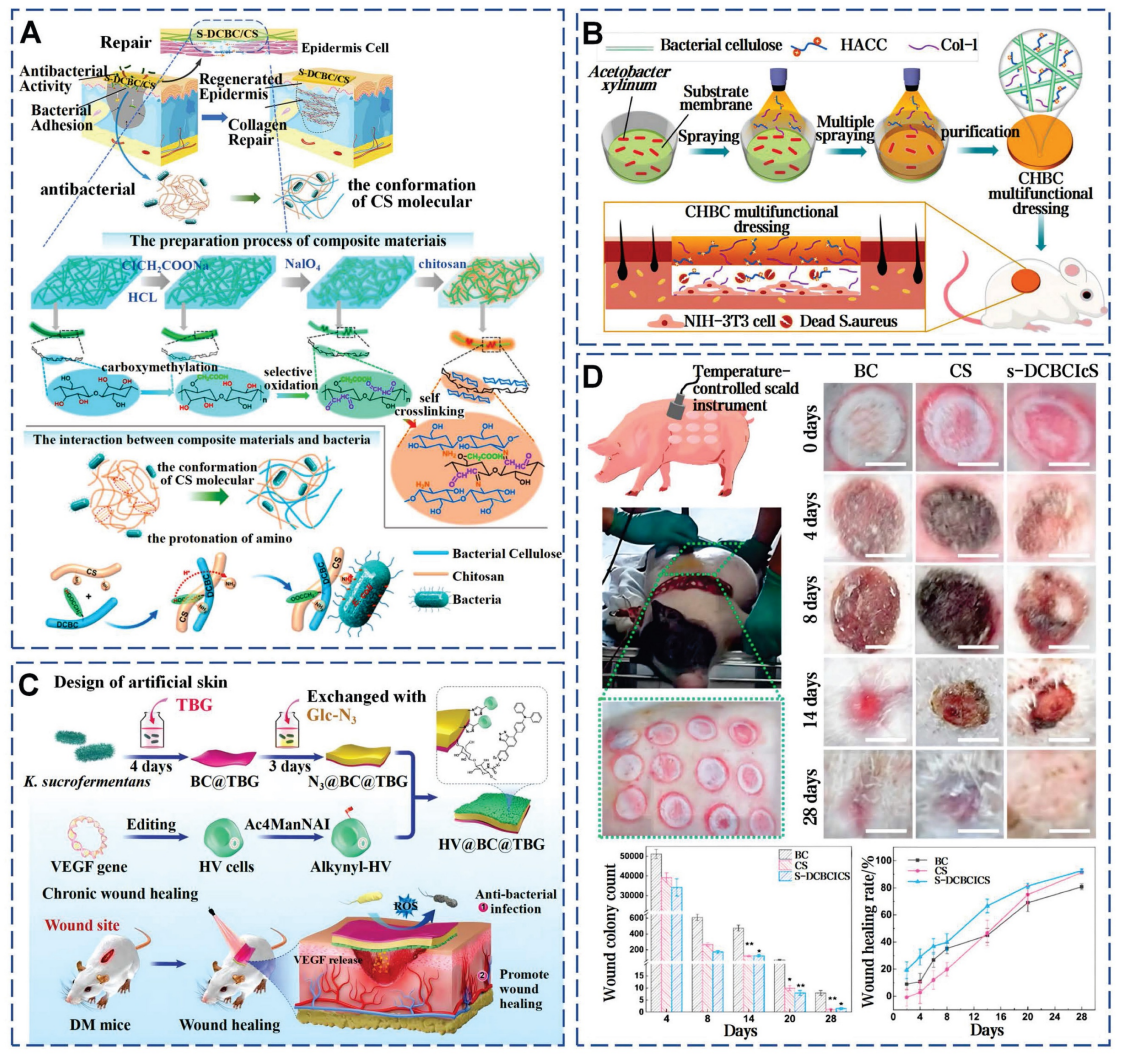
Strategies for advanced modifications of BC for infected wound healing. A) Chemical crosslinking and functional group integration in BC for enhanced wound healing. Reproduced with permission 81. Copyright 2022, Elsevier. B) HACC-infused BC dressings: integration of antimicrobial agents and bioactive compounds in BC. Reproduced with permission 82. Copyright 2021, Elsevier. C) Photothermal therapy and functional modifications of BC for complex wound healing strategies. Reproduced with permission 76. Copyright 2024, John Wiley and Sons. D) The efficacy of modified BC dressings on deep second-degree scald wounds in bama miniature pigs. Reproduced with permission 81. Copyright 2022, Elsevier.

Propolis is a natural viscous substance with multiple antimicrobial components. By incorporating Water Extract of Propolis into high-porosity polyurethane foam dressings, the antimicrobial activity of the dressings is significantly enhanced 190. The Film-forming System (FFS) is a non-solid topical drug delivery system that enables sustained release of medication, rapid drying, and good film adaptation, making it suitable for local wound healing applications. A novel FFS using propolis from the stingless bee as the active ingredient, effectively inhibits *S. aureus* and *S. epidermidis,* highlighting propolis's role in infected wound healing 191. BC has been enhanced through innovative modifications to overcome its natural antibacterial limitations. Chemical modification strategies can introduce functional groups into the side chains of BC, enhancing mechanical properties and antibacterial performance. Carboxymethylated and selectively oxidized introduced aldehyde and carboxyl groups into BC chains, achieving over 95% *in vitro* antibacterial efficacy against *E. coli* and *S. aureus* through active antibacterial effects. In a deep second-degree infected burn model of *Bama miniature* pigs, this material attains an 80% healing rate within three weeks, surpassing traditional chitosan dressings (Figure 14A) 81. Secondly, the addition of antimicrobial agents can enhance the functionality of BC. Hydroxypropyl trimethyl ammonium chloride chitosan (HACC), an antimicrobial agent derived from chitosan, exhibits superior antibacterial performance due to its cationic quaternary ammonium groups and enhanced water solubility. Through the membrane-liquid Interface culture technique, collagen I and HACC can be integrated into the network structure of BC (Figure 14B) 82. Additionally, combining with Usnic acid and Sanxan gel 78 or embedding Ag-MOF and curcumin 79 can also efficiently control bacterial infections and mitigate inflammatory reactions through the controlled release of drugs. Thirdly, combined with photothermal therapy, a novel living artificial skin HV@BC@TBG has been prepared by sandwiching the photosensitizer TBG and functional living cells HV on both sides of BC (Figure 14C) 76. This design leverages the TBG layer's ability to generate ROS under light exposure to effectively eradicate bacteria, while the HV layer functions as a living cell factory that continuously secretes VEGF, facilitating wound repair. These findings highlight that the unique microarchitecture of BC not only promotes efficient material exchange and cell penetration but also establishes a solid foundation for the tailored design and functional modification of complex wound healing strategies through precise interfacial engineering.

### 6.5. Oral mucosal defects

Oral mucosal defects, characterized by circular mountain-like defects and ulcerations, affect eating and speaking when getting severe. Among the most common oral conditions, oral mucosal defects affect over 20% of the general population 192. Oral mucosal defects are notorious for their recurrence and resistance to healing, particularly recurrent oral ulceration (ROU). Notably, no existing pharmaceuticals can fully eradicate ROU. The etiology remains unclear, with potential factors including genetics, allergies, infections, immune dysregulation, systemic diseases, microbial imbalances, nutrient deficiencies, and psychological stress 193. Current treatments, including growth factor gels, antibiotics, corticosteroids, trichloroacetic acid, and metronidazole patches, are limited by issues like short duration, secondary infections, immune suppression, mucosal damage, and side effects 194. Consequently, there is an urgent need to develop oral ulcer treatments with natural drugs that have minimal side effects and enhanced therapeutic efficacy.

**Figure 15:**
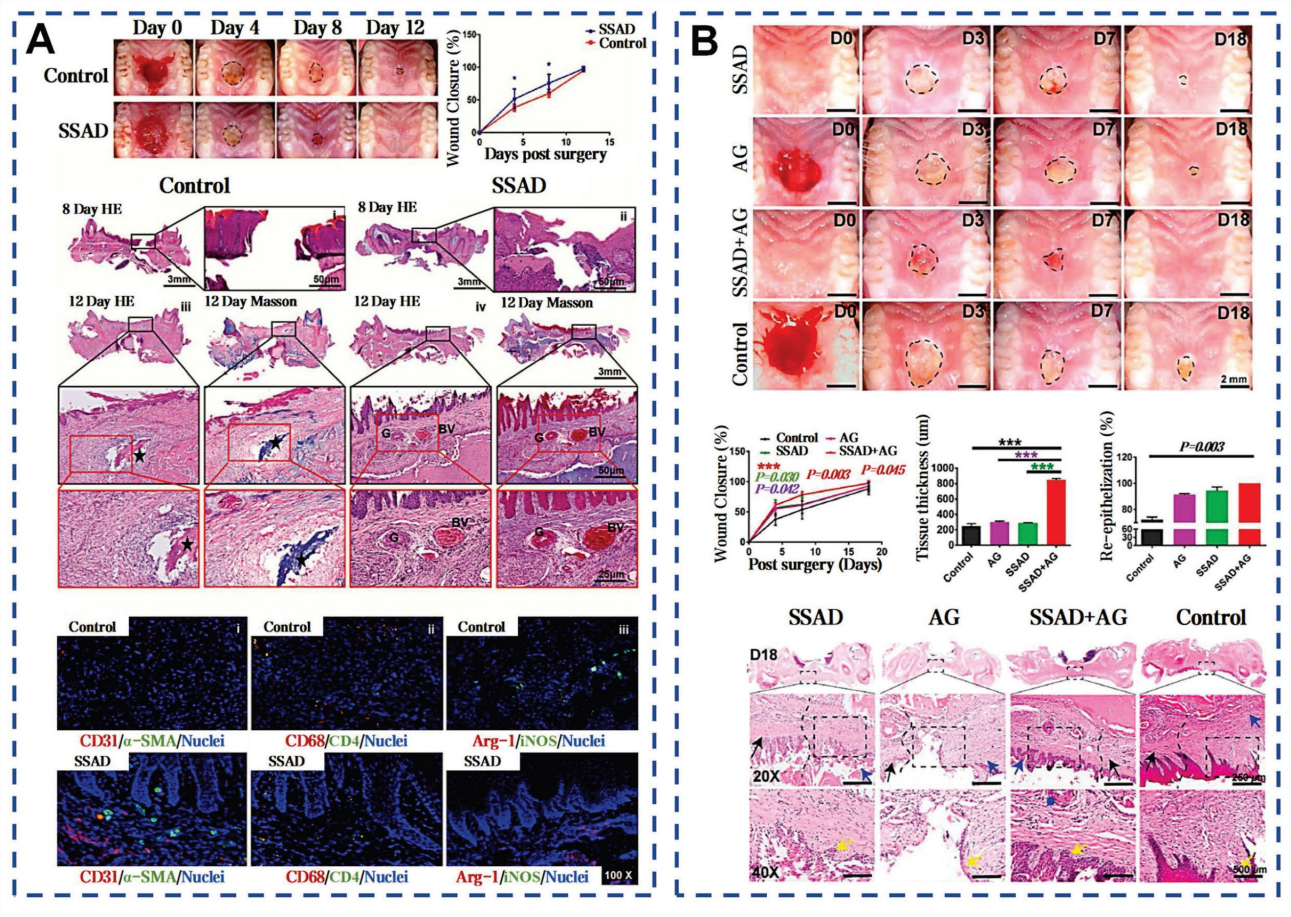
Selected cases of natural mucus utilization in oral ulcer. A) Effect of SSAD on the healing of oral palate wounds. Reproduced with permission 38. Copyright 2021, John Wiley and Sons. B) Effects of SSAD and SSAD+AG on oral palate mucosal injury in SD diabetic rats. Reproduced with permission 39. Copyright 2022, Elsevier.

SSAD powder accelerates intraoral wound healing by forming a protective barrier and promoting tissue repair. The underlying alveolar bone has no noticeable signs of necrosis, and the surface is partially covered by blood clots and degraded tissue mass in the SSAD groups (Figure 15A) 40. Its porous structure facilitates sustained drug release, enhancing therapeutic outcomes 38. Fresh aloe vera mucilage extract enhanced oral mucosal healing through immune modulation, increasing CD8+ cells and a notable increase in IL-2 and IFN-γ levels 195. The aloe mucilage also demonstrates significant antioxidant effects by increasing the activity of SOD in both plasma and mucosa and reducing malondialdehyde levels. These findings provide the first evidence of the potential of aloe mucilage to bolster innate immunity and mitigate oxidative harm, presenting a novel approach for oral ulcer therapy. Yang *et al.* performed a Meta-analysis to evaluate the clinical effectiveness and safety of propolis for treating oral mucosal defects, encompassing 23 randomized controlled trials with a total of 2467 participants 196.

The propolis treatment group exhibited a significantly higher total effectiveness rate compared to the control group. Additionally, no adverse reactions related to propolis were observed in these experiments. These findings underscore the potential of natural mucus in addressing the complex mechanisms of oral ulceration, offering promising strategies for clinical application.

## 7. Future Perspectives

Despite natural mucus demonstrates inherent therapeutic superiority, there are still many challenges demand resolution. 1) In terms of composition, fresh natural mucus often contains high water content, complicating active ingredient concentration control and posing preservation risks. 2) The abundant proteins in natural mucus can affect its viscosity and bioactivity. However, their conformations are sensitive to temperature and enzymatic degradation thereby restricting its stable application. 3) Although natural mucus shows anti-inflammatory and antimicrobial properties, the relative contributions of specific bioactive compounds remain uncharacterized. The crudely extracted natural mucus might still be unable to substitute specialized antimicrobial dressings. 4) Many animals secrete different types of mucus in response to various stimuli or different diets. Plant mucilages might also vary with seasons and environmental changes. Some microbe-derived mucus has also been proven to have its properties altered by artificially adding components. All these factors can lead to changes in the structure and composition of natural mucus, further affecting the control of standardization. We summarized the crucial properties of advanced wound dressings and the advantages and limitations of natural mucus under these indicators in Table 3.

In the future, we can envision a new era of highly personalized and precise wound treatment by integrating artificial intelligence (AI), 3D printing, and advanced bioengineering techniques. Research on natural mucus may focus on the following areas: 1) Raw material selection: Explore more biological resources, ensure sustainability, improve yield and maximize the retention of active ingredients. For natural mucus with excellent effects, it is necessary to clarify the composition ratio, structure and standardization parameters of its key components. Conduct long-term immunological and in-depth toxicological assessments to accelerate its commercialization process. 2) Structure-Function Optimization: Gene editing may be used to enhance the yield of effective ingredients in raw materials. With specialized design, the stability and processability of natural mucus can be improved through chemical modification and physical processing. Machine learning algorithms can predict optimal component ratios, accelerating rational material design. 3) Preparation method optimization: Techniques like electrospinning, nanoneedles, 3D bioprinting, and layer-by-layer assembly can be customized according to the shape of the wound. Methods like microfluidic patterning and biomimetic templating can further improve biological interaction, integration, adaptability, thereby enabling precise wound management. 4) Multi-module integrated design: Multifunctional integration without effect on the inherent therapeutic properties of natural bioadhesives represents a critical advancement. When integrated with biosensors and AI-based wound assessment platforms, wound dressings with wound monitoring, data transmission, smart response and personalized therapy may become a trend.

## 8. Conclusions

In recent years, due to the rapid progress in regenerative medicine and bioengineering technology, the high-efficiency wound healing attracts widespread attention. Natural mucus has emerged as a promising wound dressing by its innate biocompatibility, adhesion and multifaceted bioactivity. It can replicate the ECM structure while enabling physiological moisture-oxygen exchange and localized bioactive molecule delivery. Advanced fabrication technologies can enable the standing limitations to accelerate the development of natural bioadhesive. In this review, we summarized several representative natural mucus substances from animal, plant, and other complex sources. We further analyzed their applications in different types of wound management, including skin injuries, diabetic wounds, burns, infected wounds, and oral mucosal defects. These mucous substances have shown significant therapeutic effects, such as hemostasis, anti-inflammatory and antioxidant, cell growth, angiogenesis, wound closure and antibacterial properties.

## Figures and Tables

**Table 1:** Main components of natural mucus associated with wound healing and their functions

Components	Representative molecules	Primary categories	Function and mechanism	Ref.
Amino acids	Proline, lysine, cysteine, glutamic acid, aspartic acid	Structural molecules; Signaling molecules; Immunomodulatory molecules	Form ionic bonds with tissue surfaces via H-bonds/π-π stacking; Enhance viscosity, elasticity, and resistance to wound environmental factors	27
Protein	Mucins, enzymes, collagen, cytokines, antimicrobial peptides	Structural molecules; Signaling molecules; Hemostatic agents; Antimicrobial agents; Immunomodulatory molecules	Cross linked network provides shear-resistant scaffolds; Enhance cell adhesion and angiogenesis; Prevent infection	28
Polysaccharides	Glycosaminoglycans, pectin, heparan sulfate, hyaluronic acid	Structural molecules; Hemostatic agents; Immunomodulatory molecules	Responsive pH/redox adhesion and dynamic bond reorganization contribute to the hydration and lubrication of the wound site.	29
Lipids	Polyunsaturated fatty acids, phospholipids,	Signaling molecules; Immunomodulatory molecules	Integrate into cell membranes; Suppress pro-inflammatory mediators	24
Inorganic salts	Electrolytes, trace elements	Hemostatic agents; Immunomodulatory molecules	Form stable hemostatic plugs by electrostatic crosslinking with mucins; Enhance ionic interactions with wound exudate to stabilize the dressing and prevent maceration.	30
Other organic compounds	Phenolics, flavonoids, catechols, quinone derivatives, nucleic acids	Antimicrobial agents; Signaling molecules; Immunomodulatory molecules	Disrupt bacterial cell membranes and prevent infection; Scavenge ROS	28

**Table 2:** Main mechanisms of wound healing and therapeutic applications of natural mucus from diverse sources: A comprehensive overview

Type	Extraction method	Therapeutic mechanism	Representative composition	Full-thickness skin defects	Skin incision	Diabetic wound	Mucosal injury	Burn wound	Infected wound	Current progress	Ref.
*Andrias davidianus*	Non-invasive abrade skin, lyophilize, grind	Hemostasis, moisturization, barrier function, epithelialization, neovascularization, stem cell recruitment	Mucins, polysaccharides, growth factors, antimicrobial peptides, phenolic compounds, electrolytes	*+*	*+*	*+*	*+*			Laboratory research phase	37-40
*Snail*	Harvest mucus, lyophilize	Hydrophilic adhesion, M2 macrophage polarization, anti-inflammation, antioxidant, antibacterial, EGF promotion	Glycosaminoglycans, mucins, allantoin, ethanolamine, EGF-like peptides,	*+*	*+*	*+*		*+*		Laboratory research phase	12, 41-44
*Mussel*	Chemical/enzymatic extraction	Stable adhesive, cell adhesion/proliferation, coagulation, antioxidant, ECM synthesis	Mussel foot proteins, dopamine, polysaccharides, polyunsaturated fatty acids	*+*	*+*			*+*	*+*	Laboratory research phase	45-50
*Okra*	Soak seeds, filter, lyophilize	Hemostasis, antioxidant, M2 macrophage promotion, epithelial regeneration, collagen deposition	Pectin, acidic polysaccharides (galacturonic acid), flavonoids, phenolic acids	*+*		*+*				Laboratory research phase	51-55
*Aloe vera*	Separate inner gel, filter, concentrate	Fibroblast proliferation, epidermal/vascular regeneration, anti-inflammatory, antioxidant, analgesic, antibacterial	Acetylated mannans, polysaccharides, aloin, lupeol, salicylic acid, gibberellins, vitamins E/C, amino acids	*+*		*+*		*+*	*+*	Partial clinical application	56-61
*Propolis*	Solvent extraction	Collagen expression, ECM remodeling, antioxidant, anti-inflammatory, antimicrobial	Resin acids, flavonoids, terpenes, enzymes, Caffeic acid derivatives, minerals	*+*		*+*	*+*	*+*	*+*	Partial clinical application	62-73
*Bacterial cellulose* (BC)	Bacterial fermentation, collect, purify	Moisture retention, exudate absorption, physical barrier, nano-porous structure	β-1,4-glucan, trace proteins, organic acids, Exopolysaccharide matrix	*+*		*+*		*+*	*+*	Routine clinical use	74-82

**Table 3:** Summary of main critical properties for natural mucus wound dressing and their advantages and limitations

Crucial properties	Definition & clinical importance	Advantages & limitations		
		Animal mucus	Plant mucilage	Complex-sourced natural mucus
Wet adhesion and absorption capacity	Adhere to moist wound bed, absorb exudate, maintain optimal hydration and prevent maceration	Hydrophobic molecules enable direct tissue adhesion in high-exudate environment; Sensitive to temperature/pH fluctuations	High absorption of polysaccharides facilitates wet adhesion and water retention; Excessive swelling weakens adhesion; Reduced adhesion to dry and necrotic tissues	Nanofiber-wound interlock; Crosslinking reduces capacity; Require modification for enhanced adhesion
Moisture retention and oxygen permeability	Maintain hydration for autolytic debridement and cell migration, enable oxygen exchange for aerobic healing and angiogenesis	Moderate absorption for protein and lipid; Susceptible to enzymatic degradation; Long-term retention may cause collapse; High-protein exudate clogs pores	Hydrophilic matrix sustains moisture and oxygen exchange; Over-hydration risk in high-exudate environment; Reduced water retention during degradation	Elevated concentration induces hypoxia; Unsuitable for anaerobic infection control; Synthetic additives impede gas exchange and breakdown
Mechanical resilience	Resists deformation and fracture during movement	Require prolonged in situ gelation time; Relatively low strength tears during movement; Shrink upon drying, pulling wound edges	Excellent contour adaptation; High ductility when hydrated but weak tensile strength; Brittle when dehydrated	Rigid structure limits deformation
Antimicrobial activity	Inherent capacity to kill/inhibit pathogens or incorporate antimicrobial agents	Defensin and immunoglobulins provide activity; Reduced by proteases in chronic wounds	Anthraquinones and acemannan disrupt biofilms; Limited efficacy against Gram-negative strains	Species-dependent
Immunomodulation	Modulates inflammatory cytokines to prevent chronic inflammation	Risk of allergy and immune reactions from exogenous factors	Effects may be inconsistent or paradoxical at high concentrations	Potential endotoxin contamination
Biocompatibility	Non-toxic, non-irritating, non-bioaccumulative, and non-allergenic to surrounding tissue	Zoonotic pathogen transmission risk; Potential allergens	Generally low immunogenicity; Residual pesticides and extraction solvents may cause reactions	Context-dependent
Ease of application and removal	Applicable and removable without causing pain or tissue damage	Require temperature control; Residue risk from fragments;	Brittle films may fragment during removal; potential residue	Without concentration processing; Complex applications require professional handling
Production feasibility	Sustainable source, cost-effectiveness, scalability, sterilization compatibility, regulatory pathway	High-cost live harvesting; Expensive medical-grade purification; Sterilization and storage escalate expenses	Renewable farming sources; Seasonal batch variability impedes standardization	Require equipment investment; Rigorous filtration needed for particulate residues
Other considerations	Odor control, pain management, eco-friendliness	Sulfur and amine components yield distinctive odors; Neuroinflammatory reaction risk	Cooling sensation reduces burning; Peculiar odor when improperly preserved	Degradation often requires multiple enzymes; Added complexity affects degradation

## Abbreviations

ANFMs: Aloe nanofiber membranes
ADENHs: Aloe-derived exosome nanoparticles
BC: Bacterial cellulose
bFGF: Basic fibroblast growth factor
CAPE: Coffee acid phenethyl ester
CAs: Cyanoacrylates
d-SMG: Dried snail mucin gel
ECM: Extracellular matrix
EE: Earthworm mucus extract
EGF: Epidermal growth factor
EPS: Extracellular polymeric substances
FGF: Fibroblast growth factor
FFS: Film-forming System
GAGs: Glycosaminoglycans
GM-CSF: Granulocyte-macrophage colony-stimulating factor
HACC: Hydroxypropyl trimethyl ammonium chloride chitosan
HGF: Hepatocyte growth factor
HUVECs: Human umbilical vein endothelial cells
IL-1: Interleukin-1
IL-6: Interleukin-6
Mfps: Mussel foot proteins
NIR: Near-infrared
NF-κB: Nuclear factor kappa-light-chain-enhancer of activated B cells
OPS: Okra polysaccharides
OHG: Okra hydrogel
PDGF: Platelet-derived growth factor
PNPs: Propolis nanoparticles
PUFAs: Polyunsaturated fatty acids
ROU: Recurrent oral ulceration
ROS: Reactive oxygen species
SAGs: Skin secretion-derived glycosaminoglycans of *Andrias davidianus*
SNM: Snail mucus
SOD: Superoxide dismutase
SSAD: Skin secretions of *Andrias davidianus*
TGF-β: Transforming growth factor-β
TNF: Tumor necrosis factor
VEGF: Vascular endothelial growth factor
